# The Direct Mechanocatalytic Suzuki–Miyaura Reaction of Small Organic Molecules

**DOI:** 10.1002/anie.202205003

**Published:** 2022-07-13

**Authors:** Wilm Pickhardt, Claudio Beaković, Maike Mayer, Maximilian Wohlgemuth, Fabien Joel Leon Kraus, Martin Etter, Sven Grätz, Lars Borchardt

**Affiliations:** ^1^ Inorganic Chemistry I Ruhr-Universität Bochum Universitätsstraße 150 44801 Bochum Germany; ^2^ Deutsches Elektronen-Synchrotron (DESY) Notkestraße 85 22607 Hamburg Germany

**Keywords:** Ball Milling, Cross-Coupling, Direct Mechanocatalysis, Mechanochemistry, Transition-Metal Catalysis

## Abstract

The molecular Suzuki cross‐coupling reaction was conducted mechanochemically, without solvents, ligands, or catalyst powders. Utilizing one catalytically active palladium milling ball, products could be formed in quantitative yield in as little as 30 min. In contrast to previous reports, the adjustment of milling parameters led to the complete elimination of abrasion from the catalyst ball, thus enabling the first reported systematic catalyst analysis. XPS, in situ XRD, and reference experiments provided evidence that the milling ball surface was the location of the catalysis, allowing a mechanism to be proposed. The versatility of the approach was demonstrated by extending the substrate scope to deactivated and even sterically hindered aryl iodides and bromides.

## Graphical abstract

Utilizing polymer milling vessels and liquid‐assisted grinding, direct mechanocatalytic Suzuki coupling was conducted successfully without abrading the palladium catalyst. Various target molecules were synthesized in good yields within 1 h. In situ XRD and parameter studies provided deep insight into the mechanism of direct mechanocatalysis.

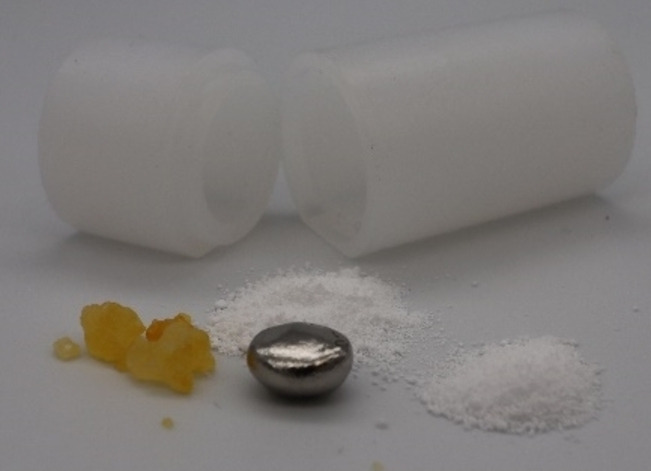

## Introduction

Mechanochemistry is considered to be among the most promising methods regarding green chemistry.[^1^, ^2^] It reduces the waste of a chemical reaction and thus their environmental burden by eliminating the use of solvents entirely.[^2^, ^3^] Over the last decades, numerous classical liquid‐phase C−C coupling reactions were performed successfully under solvent‐free mechanochemical conditions.[^4^, ^5^, ^6^, ^7^, ^8^] Amongst them are many Pd‐catalyzed reactions such as the Sonogashira, Suzuki–Miyaura, or Heck reaction.[^5^, ^8^, ^9^, ^10^, ^11^, ^12^] In general, sophisticated ligand systems can be omitted since the catalyst solubility is no longer required under mechanochemical conditions.^[9]^ Although simple Pd salts such as Pd^II^ acetate can be used, those still contribute decisively to the cost of the system. Under economic and ecological consideration, their reuse is crucial, yet complicated and time‐consuming and thus mostly omitted in academic research, where the catalyst is usually discarded after the reaction.[^5^, ^6^, ^13^] A concept that overcomes this issue is direct mechanocatalysis.[^4^, ^14^, ^15^] Here, the milling ball serves as catalyst itself. During milling, i.e. during the collision of the milling ball with the milling vessel or with other milling balls, the substrates react heterogeneously on the surface of the milling ball. The catalyst separation and recyclability are as easy as taking the milling ball out of the reaction mixture and reusing it in the next reaction.^[15]^ So far, only a few reactions such as cyclotrimerizations, transition metal‐catalysed couplings and hydrogenation reactions are known to proceed under direct mechanocatalytic conditions.[^16^, ^17^] High abrasion of the costly catalytically‐active balls during milling impedes pharmaceutical application as strict limits on metal contaminants during the production process are given.[^4^, ^18^] Finally, longer reaction times compared to classical metal salt‐catalysed protocols are necessary.[^6^, ^18^] Thus, the disadvantages of this novel catalysis concept are outweighing its advantages currently, severely limiting a widespread application of this promising technique.

In this contribution we present the first Suzuki cross‐coupling under direct mechanocatalytic conditions with yields rivalling those of homogeneous catalysis. We were able to perform the Suzuki coupling using a wide array of substrates, without solvents and most importantly, with palladium abrasion below 1 μg in as little as 60 min. This work provides a direct mechanocatalytic protocol to modern chemistry as an enticing alternative for established solid‐state and solution‐based protocols alike.

## Results and Discussion

**Figure 1:**
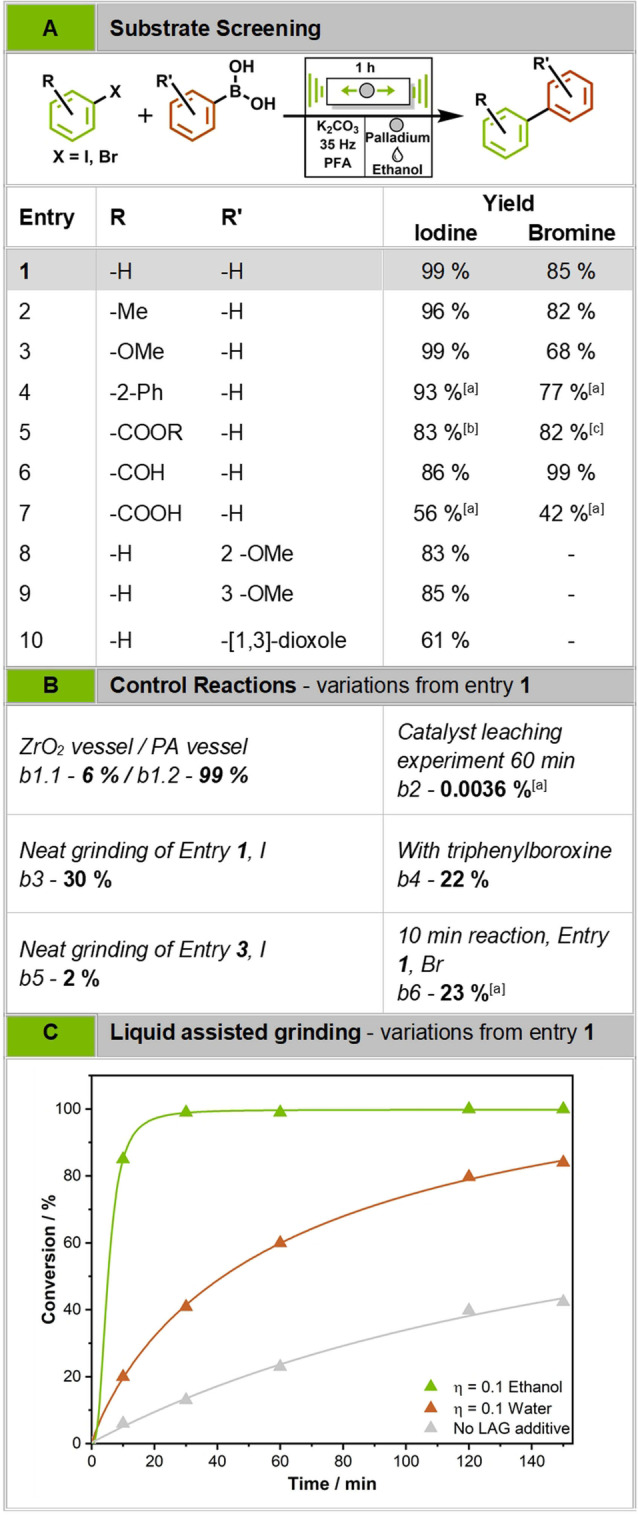
Summary of the performed substrate screening (A) and control experiments (B). All reactions were performed in 19 ml PFA vessels at 35 Hz for 1 h with ethanol as LAG additive. Layout adapted from ref. [7b]. C) Time‐ dependent GC‐MS conversion. After each time step, the reaction was stopped, and a GC sample was taken. This observation showed that LAG additives play a major role in the reaction, with alcohols being even more efficient than water. Even after 16 h of milling, the reaction without any additional liquid did not surpass 50 % yield.^[19]^ [a] Yield determined by HPLC. [b] 4‐Iodo‐methyl benzoate was used. [c] 4‐Bromo‐ethyl benzoate was used.

As model system, we chose the reaction of phenylboronic acid and iodobenzene. We took a typical mechanochemical reaction protocol for Suzuki cross‐coupling known from literature and adapted it to direct mechanocatalysis.^[14]^ It involved one 10 mm palladium milling ball of approximately 4 g, 1 mmol of each starting material and 1 g of K_2_CO_3_ in a 10 mL zirconia milling vessel. After 1 h of milling at 35 Hz, a yield of 6 % was achieved while 119 mg palladium were abraded (cf. Figure 1B, control reactions b1.1). Abrasion had to be avoided and the reaction rate had to be increased to achieve high yields in acceptable reaction times. Abrasion is caused by two different events, that is ball‐ball and ball‐vessel collisions. While the former can be tackled by using only one single milling ball, the latter is mainly impacted by the hardness of the milling vessel material. Common vessel materials applied in mechanochemistry such as ZrO_2_, WC or steel feature an excessive hardness as compared to the Pd ball.^[15]^ In conventional mechanochemical reactions, one milling ball would be used in a vessel made from zirconia or steel of about 10 mL volume.[^9^, ^10^, ^11^] Using such vessels with one Pd milling ball, however, leads to inacceptable abrasion. Using two Pd balls would lead to even more abrasion upon collision of the two palladium balls.^[14]^ Therefore, we utilized 19 mL polymer milling vessels from poly (methyl methacrylate) (PMMA), polytetrafluoroethylene (PTFE), and perfluoralkoxy alkane (PFA). They can easily be manufactured by CNC mills or by lathe turning, usually to much lower costs than commercially available milling vessels made of ZrO_2_ or steel. While PMMA and PTFE turned out to be unsuitable due to their lacking chemical or mechanical resistance, respectively (cf. Supporting Information Table S1), PFA has proven to sustain the milling conditions while being translucent, which enables in situ studies and thus was utilized in this study (cf. Supporting Information Chapter 2.1). However, since fluorinated polymers are an environmental concern, the use of this vessels should be critically questioned. To address this issue, we reproduced the experiments in a vessel made from inexpensive, non‐toxic, bio‐based polyamide‐6 and observed equivalent results for the optimized reaction protocol. (cf. Supporting Information Table S2, Figure 1 Entry b1.2).

After establishing a suitable milling system, we quickly realized that our standard conditions never surpass 50 % conversion even after 16 h of milling as time‐dependent GCMS measurements show (cf. Figure 1C, no LAG additive, Supporting Information Table S3). However, we noticed an unexpected signal in the GC‐MS (cf. Supporting Information Figure S3, 1), which corresponds to the condensation of three units phenylboronic acid to a boroxine trimer. We suspected this species to be less reactive in the coupling reaction under ball milling conditions and confirmed this by utilizing the trimer as starting material instead of the boronic acid (cf. Figure 1B, control reactions b3+b4). To avoid the formation of the boroxine, which is formed in equilibrium under elimination of water,^[20]^ liquid additives were added to shift the equilibrium towards the monomeric phenylboronic acid. Adding small quantities of liquid additives is described in mechanochemistry as liquid‐assisted grinding (LAG). A fraction of the substrate mass, described by *η*, is added to the reaction mixture. This fraction typically differs between *η*=0–2 μL mg^−1^.^[21]^ Indeed, the addition of *η*=0.1 water enhanced the conversion, from 30 to 60 % yield after 1 h of milling (cf. Figure 1C) and the signals of boroxine were no longer observed in the GC‐MS (cf. Supporting Information Figure S3, 2). If instead *η*=0.15 ethanol was utilized the same reaction time led to quantitative yields and even after as little as 10 min 85 % of the product could be isolated (cf. Figure 1C). These additives thus counter the formation of the unwanted boroxine, either due to the formation of phenylboronic ethyl ester as it was confirmed by Raman spectroscopy (cf. Supporting Information Chapter 4.3) or by influencing the equilibrium of the boroxine formation. Additionally, although phenylboronic acid esters are known to react slower in conventional Suzuki coupling,^[22]^ they are beneficial in the direct mechanocatalytic approach for one reason—they are liquid. Before utilizing LAG, we could already observe that the reaction proceeds faster for liquid substrates as compared to solid ones. If no LAG is applied, the reaction of iodobenzene (liquid) yields 30 % of product after 1 h (cf. Figure 1B, control reaction b3) compared to 4‐iodoanisole (solid) which leads to less than 5 % despite being activated for the initial oxidative addition step.^[23]^ Moreover, the most efficient LAG additives contain hydroxy groups as they can interact with the phenylboronic acid (Supporting Information Chapter 2.4). Other solvents, which are capable of dissolving the starting materials, but are not able to form phenylboronic esters such as acetone or ethyl acetate, lead to even worse results than the sample without LAG (Supporting Information Chapter 2.4), confirming that conversion of the phenylboronic acid into an ester is of major importance. Please note, LAG amounts used during these experiments refer to liquid‐substrate ratios of 0.1–0.15, meaning the liquid cannot dissolve the substrates entirely, it rather provides a diffusion‐enabling boundary layer on the ball/powder and increases the mass transport therein.

**Figure 2:**
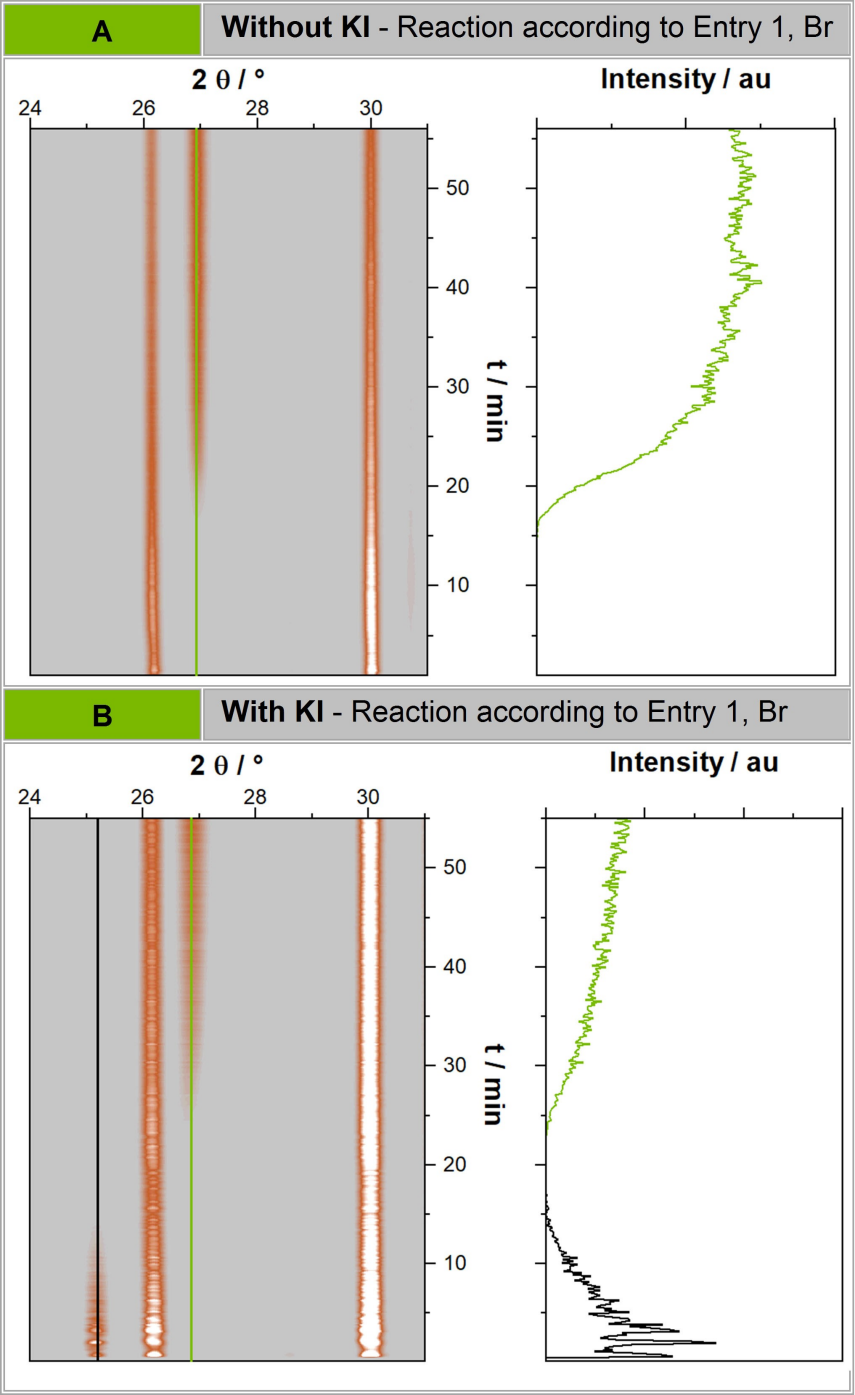
In situ PXRD analysis of the coupling between bromobenzene and phenylboronic acid. A) Without the addition of potassium iodide. Green lines highlight the new reflections after the product is formed. B) The coupling between bromobenzene and phenylboronic acid with the addition of 10 mol% potassium iodide. Added potassium iodide is represented by the black line. Most noticeably is the consumption of the added KI and the subsequent, delayed product formation. This shows a slower kinetic of the reaction with KI additive, even though the reaction results in a higher yield.

We further investigated the reactivity of different halide species in the direct mechanocatalytic Suzuki coupling. Brominated substrates convert significantly slower than iodinated (cf. Figure 1A). For instance, iodobenzene with ethanol as LAG additive yields 99 % coupling product after 1 h, while bromobenzene does not surpass 63 % in the same reaction time. To tackle this, an in situ trans‐halogenation approach was attempted. For this purpose, potassium iodide was added to the reaction mixture in order to mechanochemically exchange the bromine substituent for the more reactive iodine atom.^[24]^ This technique enhanced the reactivity of brominated compounds, enabling us to couple bromobenzene with a good yield of 86 % after 1 h of milling. In situ XRD measurements were performed using the standard reaction (cf. Figure 1A, Entry 1 Br) with 10 mol‐% potassium iodide added. We observed an initial consumption of potassium iodide before the product formation is observable (cf. Figure 2B). This confirms our finding that aryl bromides can be converted into iodo derivatives under ball milling conditions (cf. Supporting Information Chapter 4.5). Arylchlorides, however, did not show any conversion during these tests (cf. Supporting Information Chapter 4.2). Using a 10 mm Pd milling ball, ethanol (*η*=0.15) as LAG additive and in case of brominated compounds 10 mol.‐% potassium iodide at 35 Hz milling frequency for 1 h, numerous starting material combinations were tested (cf. Figure 1). Several activated, liquid aryliodines react readily and quantitatively under the presented conditions (cf. Figure 1A, Entries 1+2). To our delight, even sterically hindered starting materials such as 2‐iodobiphenyl and 2‐bromobiphenyl could be coupled with phenylboronic acid in excellent or good yields, respectively (cf. Figure 1A, Entry 4). Furthermore, substrates of varying electron withdrawing or donating character such as anisoles, carboxylic acids or carboxylic ester derivatives can be coupled in excellent to satisfactory yields rivalling those of classical liquid phase synthesis (cf. Figure 1A, Entry 5–10).

To fully utilize the potential of this approach, however, a deeper understanding of the underlying principles is necessary. Combining this enhanced LAG reaction rate with previous observations made in direct mechanocatalytic reactions we formulated two major mechanistic hypotheses:

1. The reaction is taking place on a thin film around the point of impact.
2. The palladium on the surface of the milling ball is the catalytically active site.

The first hypothesis is supported by our observations outlined above and also by calculations, which reveal that the reaction rate is too high for only one adsorbed monolayer around the whole ball, as assumed by heterogeneous catalysis, to be converted with each collision (for detailed calculation cf. Supporting Information Chapter 4.4). The substrates must accumulate in a film or multilayers around the milling ball rather than an assumed monolayer. That is why LAG provides such a significant impact. Without LAG, the film can only be formed partially for liquid substrates, while no diffusion enabling film is formed for solid substrates, explaining the reason behind the difference of solid vs. liquid substrates conversion (cf. Figure 1B, control reactions b3+b5). Upon collision between the milling ball and the vessel, this film is exposed to mechanical stress. Providing close contact and hence forcing them to react in the intended way.

In order to systematically prove the second hypothesis, i.e., catalysis being driven by the milling ball surface itself, we first need to exclude the possibility that abraded palladium nanoparticles catalyse the reaction. Therefore, catalyst leaching experiments were conducted. In one setup, the Pd ball was pre‐milled with the bulking material and the LAG reagent in absence of the substrates. This process simulates a typical reaction and should hypothetically abrade catalytically active nanoparticles. Afterwards the Pd ball was exchanged with an inert milling ball of roughly the same size and weight and the substrates were added to the mixture. Then, the milling was continued for the same time as the pre‐milling. This experiment was conducted for 10 and 60 min, respectively. However, even after 60 min of milling, only traces of the product could be found by HPLC (cf. Figure 1B, control reaction b2). In a second setup, all starting materials and the Pd ball were present in the vessel from the beginning and were milled for 2 min before the Pd ball was exchanged by an inert zirconia milling ball. If Pd nanoparticles are formed by etching processes of the starting materials, they would remain in the milling mixture and lead to an increase in yield when the milling is continued. This experiment showed that the conversion rate drops drastically as soon as the Pd milling ball is removed from the milling vessel (cf. Supporting Information Chapter 4.1.1). Thus, we conclude that the reaction does not proceed on abraded particles in the reaction mixture. We further quantified the abraded Pd by ICP‐OES measurements, confirming that below 1 ppm (900 ng) of palladium are abraded into the reaction mixture during these 60 min of milling (cf. Supporting Information Chapter 4.1.4). This value is also in line with the strict limits of elemental impurities in pharmaceutical protocols.^[25]^

**Figure 3:**
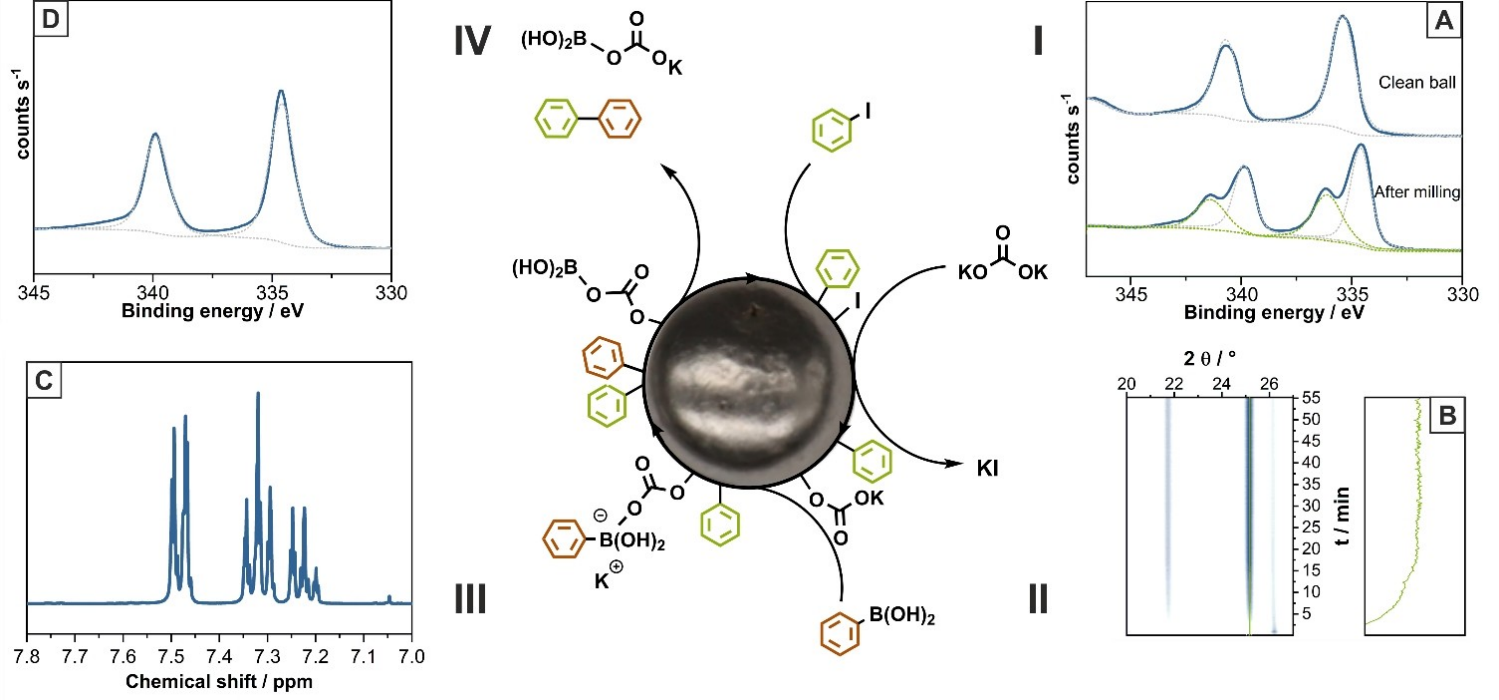
Postulated catalytic cycle on the milling ball during direct mechanocatalysis based on XPS, in situ XRD and ex situ NMR spectra. I) The oxidative addition of the aryl halogen forms a Pd^II^ species, which cannot be found on a clean Pd milling ball (A). II) The base, in this case potassium carbonate, substitutes surface‐ bound iodide by the base residue while eliminating KI, which was proven by in situ XRD (B). This base residue enables the phenylboronic acid to interact with the surface, which was not possible without the base residue, comparable to the interaction of the phenylboronic acid with the LAG additive ethanol. III) The phenylboronic acid undergoes transmetalation before IV) elimination of the desired product (C) and the boronic acid side product with subsequent restoration of the catalytic active Pd surface (D).

After these experiments, we turned our focus to the catalyst ball itself. In order to proof that the reaction is happening on the milling ball surface, we conducted XPS analysis of the milling ball before and after milling with iodobenzene (Figure 3A, Supporting Information Chapter 4.1.2). In line with our theory, we could demonstrate that palladium on an unused milling ball is found in the oxidation state Pd^0^, but changes partially to Pd^II^, which, as several reference experiments show, can only be observed if there is a) active milling and b) if aryl halides are present (cf. Figure 3, Supporting Information Chapter 4.1.2). These results were further confirmed by low vacuum SEM and EDX analysis that show adsorbed iodobenzene on the milling ball surface after milling (cf. Supporting Information Chapter 4.1.3). These observations are in accordance with the results of Sajiki et al., who investigated the mechanism of the heterogeneous catalyzed Suzuki reactions on Pd‐clusters.^[26]^ We further monitored the reaction kinetics of our reaction by synchrotron in situ X‐ray diffraction (XRD) (cf. Figure 2, Supporting Information Chapter 4.2) and followed the reaction kinetics. Although the reaction product biphenyl forms amorphously, we could track the reaction due to the formation of the highly crystalline side products (potassium halides) and the decrease of potassium carbonate. After 15 and 20 min, respectively, the first crystalline side product is observed when bromobenzene and phenylboronic acid are used (cf. Figure 2A). Please note, however, that the reaction already occurs before but yields amorphous products. This is proven by ex situ analysis, showing 23 % yield after 10 min of milling (cf. Figure 2B, control reactions b6). With the data collected during these investigations, we are also able to postulate a mechanism for the direct mechanocatalytic Suzuki coupling on the example of the coupling of iodobenzene and phenylboronic acid (cf. Figure 3).

In the beginning of the catalytic cycle (Figure 3, I), upon impact of the milling ball, the Pd surface is showing a strong interaction with the iodobenzene partially forming Pd^II^ species (cf. Figure 3A). These shifts of the Pd signals in the XPS spectra are matching the behavior of Pd‐clusters in heterogeneous catalysis.[^27^, ^28^] At the same time, milling of phenylboronic acid, potassium carbonate or a mixture of both under the same conditions does not lead to any observable interaction with the Pd ball (cf. Supporting Information Figure S6 E and F). Furthermore, we can rule out a radical pathway as it would lead to side products by radical recombination with different substitution patterns. The absence of these side products (cf. Figure 3C) in our studies leaves the oxidative addition in a concerted pathway as the only reasonable option, as it is already known for the homogeneous and heterogeneous Suzuki coupling.^[29]^ In situ XRD analysis further show the quick formation of potassium halogens after the milling is started (cf. Figure 3B, Supporting Information Figure S9A). This formation is caused by an exchange of the halogen from the milling ball surface with a residue from the base, containing an oxo group.

In the second step of the cycle (cf. Figure 3, II), the oxo binding site is capable of reacting with the phenylboronic acid as already described in literature.^[28]^ This oxo species is required as an anchor point for the boron in order to activate the boron‐bound phenyl ring, a behavior already observed during the LAG testing (cf. Figure 3, III). Since none of these boronic acid groups can be found on the milling ball of the reaction mixture (cf. Supporting Information Figure S6 E), this and the following steps are rather fast and the oxidative addition of the aryl halogen is likely the rate‐determining step for the catalytic cycle.

After the binding of the phenylboronic acid, a directed recombination of the phenyl ring of the phenylboronic acid and the former iodobenzene can occur. The high selectivity of this conversion is rooted in this step, as both substrates are introduced into the catalytic cycle by different, interdependent processes, preventing an undirected recombination of either of the substrates. An elimination of the desired cross‐coupling product (cf. Figure 3, IV) and the boronic acid derivative sets free the product as well as the side product. As the milling itself leads to a constant shedding of the boundary‐layer, both products are quickly removed from the milling ball surface, providing a vacancy so the catalytic cycle can start again with a refreshed Pd^0^ surface (cf. Figure 3D). To exclude the possibility of an aryl halide or aryl boronic acid homocoupling, a reaction of iodobenzene and 4‐methoxyphenylboronic acid was followed by XPS and GC‐MS proving that the biphenyl derivatives arise from a cross‐coupling (cf. Supporting Information Chapter 4.6).

**Figure 4:**
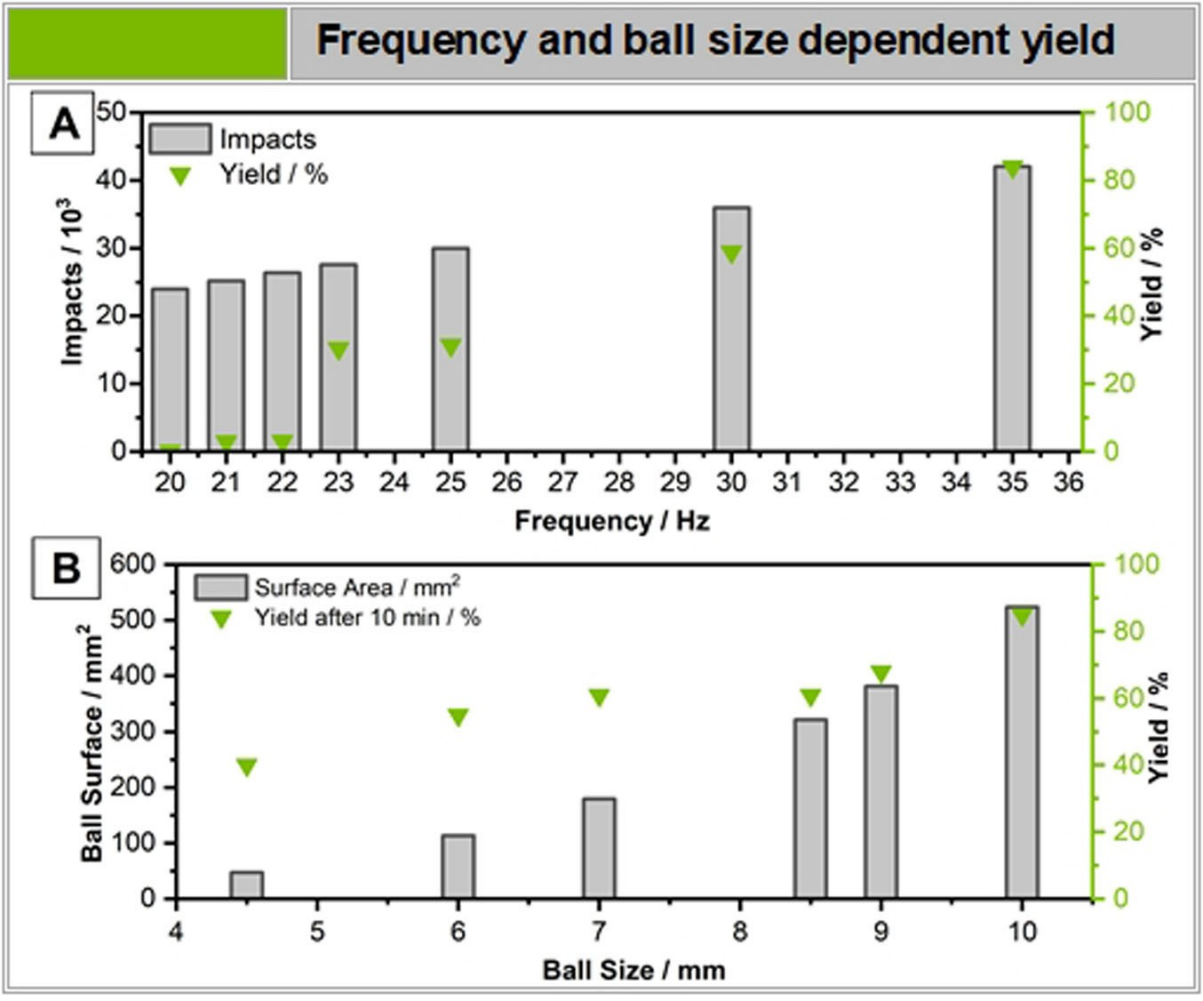
Suzuki coupling of iodobenzene and phenylboronic acid. A) The yield is shown in correlation to the frequency, ball mass and diameter. B) Milling frequency dependent yield of the Suzuki coupling. Below 23 Hz, no product formation is observable. Yield was determined by HPLC.

Deeper investigations into the mechanochemical parameters of the reactions, with an emphasis of apparent activation energy of the aryl iodine interaction, revealed the existence of an apparent threshold milling frequency (cf. Figure 4A). Below 23 Hz, only traces of product can be found. Upon further inspection the movement of the milling ball in relation to the vessel seems to be the decisive factor. While at low frequencies the ball is mostly rolling on the vessel surface, frequencies at 23 Hz and above, the milling ball detaches from the vessel surface and hits the walls in‐flight, allowing for a more effective energy transfer and higher yields (cf. Figure 4B). However, the yield does not scale linearly with the number of impacts after this threshold. A reaction conducted at 23 Hz for a set number of impacts (42k, 47 % yield) leads to less product than a reaction performed at 35 Hz for the same number of impacts (42k, 84 % yield) (cf. Supporting Information Chapter 2.5).

To confirm this further, we conducted the standard reaction (cf. Figure 1A, Entry 1, I) with palladium balls of varying size at 35 Hz. No threshold weight, was observable, as even very small and therefore light milling balls lead to yield (cf. Figure 4, B). A 4.5 mm milling ball at 35 Hz has an approximated kinetic energy of 0.685 mJ and leads to a yield of 40 %. A milling ball of 10 mm at 20 Hz has an even higher kinetic energy of approximately 2.09 mJ and a five times higher surface area, but does not lead to the desired product. That means the milling ball movement, i.e. in‐flight vs. rolling, is more important than the kinetic energy of the individual moving milling ball.

## Conclusion

In summary, with the presented protocol direct mechanocatalytic Suzuki cross‐coupling can now be performed without any abrasion on numerous substrates utilizing inexpensive polymer vessels. The standard reaction (cf. Figure 1A, Entry 1, I) yields 85 % of product after 10 min and quantitative yield after only 30 min of milling. Thereby, liquid‐assisted grinding is the game changer enhancing the reactivity greatly. Various challenging substrate combinations can be converted, even benzoic acid derivatives, which commonly feature solubility problems. In addition, an in‐depth study of milling parameters and the utilization of advanced characterization techniques shed light onto the principals of direct mechanocatalysis, proving that the place of the catalysis is indeed the milling ball surface. On the basis of these results we also proposed a mechanism for the direct mechanocatalytic Suzuki cross‐coupling. With this protocol, direct mechanocatalysis proves itself as an imaginable alternative to other, liquid or catalyst salt‐based approaches.

## Conflict of interest

The authors declare no conflict of interest.

1

## Supporting information

As a service to our authors and readers, this journal provides supporting information supplied by the authors. Such materials are peer reviewed and may be re‐organized for online delivery, but are not copy‐edited or typeset. Technical support issues arising from supporting information (other than missing files) should be addressed to the authors.

Supporting Information

## Data Availability

The data that support the findings of this study are available from the corresponding author upon reasonable request.
