# Supplementary material for: The Direct Mechanocatalytic Suzuki–Miyaura Reaction of Small Organic Molecules
Source: Angew Chem Int Ed Engl. 2022 Jul 13;61(34):e202205003. doi: 10.1002/anie.202205003 (PMC9543434; doi:10.1002/anie.202205003)
Supplement: Supplementary file 1 — Supporting Information [file ANIE-61-0-s001.pdf]

Angewandte Chemie
Eine Zeitschrift der Gesellschaft Deutscher Chemiker

Supporting Information

The Direct Mechanocatalytic Suzuki–Miyaura Reaction of Small Organic Molecules

*W. Pickhardt, C. Beaković, M. Mayer, M. Wohlgemuth, F. J. L. Kraus, M. Etter, S. Grätz, L. Borchardt**

Table of Contents

Table of Contents **2**
1. Experimental section **3**
1.1 Used equipment and characterization techniques 3
2. Milling parameters **4**
2.1 Comparison of milling material hardness 4
2.2 Multiball approach 4
2.3 Time dependent milling 5
2.4 Liquid-assisted grinding 6
2.5 Frequency dependence 7
2.6 Influence of LAG on the 2,4,6-triphenylboroxin formation 7
2.7 Milling ball recycling 8
3. Synthetic procedures **8**
3.1 Direct mechanocatalytic Suzuki coupling 8
3.2 Product characterization data and figures 9
4. Mechanistic considerations **11**
4.1 Determining the place of the catalysis 11
4.1.1 Leeching experiments 11
4.1.2 X-ray photoelectron spectroscopy 12
4.1.3 Scanning electrode microscopy and Energy-dispersive X-ray spectroscopy 14
4.1.4 Inductively coupled plasma atomic emission spectroscopy 16
4.2 In situ XRD 16
4.3 Raman investigations on the phenylboronic ester formation 18
4.4 Detailed calculations on monolayer hypothesis 19
4.5 Transhalogenation 20
4.6 cross-coupling validation 21
Author contributions **23**

1. Experimental section

1.1 Used equipment and characterization techniques

General. All reagents were obtained from commercial suppliers at least in synthesis grade purity and were used without further purification. Organic solvents used for LAG were obtained in analysis grade. Water used for LAG was taken from a tap in deionized form. The milling was carried out exclusively in a Retsch MM-500 vario ball mill. The milling vessels were custom made from PFA and PTFE. The raw material for the vessels were obtained from Technische Materialien in Görlitz, Germany.

Raman spectra were obtained using a RENISHAW inVia Qontor Raman microscope with 50x object (NA = 0.50, 8.2mm free working distance). The wavelength for the measurement was 785 nm with 0.5 to 10 % laser power dependent on the sample.

Gas chromatography-mass spectrometry (GCMS) was performed on a Shimadzu NEXIS 2030 gas chromatograph with a 30 m 0.25 mm ID, 5 % Diphenyl / 95 % dimethyl polysiloxane column. Electron ionization with an injection temperature of 200 °C was used. 1 mg of crude product was dissolved in 1 mL of DCM, filtered through a cotton filter and transferred to a GC vial.

High performance liquid chromatography (HPLC) was conducted on a Shimadzu Nexera LC-40 lite. A Nucleodur C18, 3 µm Reversed phase column from Machery-Nagel was used as stationary phase. An isocratic solvent mixture consisting of 75 % acetonitrile and 25 % water at a flowrate of 0.7 mL/min was used as mobile phase. 3-5 mg of crude product were dissolved in 3 mL of acetonitrile/water mixture (60 : 40), filtered through a syringe filter and transferred to a HPLC vial.

In-situ XRD analysis Two-dimensional PXRD images were collected at the DESY/PETRA III beamline P02.1 using a Perkin Elmer XRD 1621 flat panel detector consisting of an amorphous Si sensor equipped with a CsI scintillator (pixel number 2048 x 2048, pixel size 200 x 200 µm²). Diffraction images were subsequently integrated in one-dimensional PXRD patterns with the DAWN Science package using a wavelength λ = 0.20733 nm. For creating a 2D figure, integrated diffraction patterns were baseline corrected with Sonneveld-Visser algorithm.

Scanning Electrode Microscopy and Energy-dispersive X-ray spectroscopy (SEM+EDX).
SEM images were recorded using a high-resolution scanning electron microscope (JEOL JSM-IT800) at 15 kV. An Oxford *Ultim Max* Silicon Drift Detector (SDD) at 15 kV was used as primary detector.

X-ray photoelectron spectroscopy (XPS). XPS Spectra were recorded on a Nexsa G2 Surface Analysis System. X-ray source type: Monochromated, micro-focused, high-efficiency Al Kα X-ray source. The spectra were obtained by scanning 20 times with 50 keV. Analyzer type: 180°, double-focusing, hemispherical analyser with a 128-channel detector

Inductively coupled plasma atomic emission spectroscopy (ICP-OES). ICP-OES measurements were performed on an Analytikjena Plasma Quant PQ 9000. Samples were taken directly from the milling vessel after the milling, dissolved in an acetic acid acidified mixture of acetonitrile and water (60 : 40).

X-ray fluorescence spectroscopy (XRF). The XRF-spectra were obtained by a Vanta™ Handheld XRF Analyzer with a 40 keV Rhodium X-ray tube.

Milling balls. Palladium milling balls and polymer milling balls were obtained from SMT Metalle Wimmer in Weinburg, Austria. Due to the manufacturing process of these milling balls (arc sintering), they are not perfectly spherical but flattened. The diameter is measured at the broadest part of the milling ball. Polymer milling balls were obtained from Kugel-Winnie in Bamberg, Germany.

2. Milling parameters

2.1 Comparison of milling material hardness

Table S1: Possible milling materials with their hardness and density.

Material	Hardness (scale)	Density [g/cm^3]
**POM**	81 (Shore)	1.41
**PFA**	62 (Shore)	2.15
**PTFE**	55-60 (Shore)	2.15
**PMMA**	87-88 (Shore)	1.18
**Polyamide-6**	75.0 - 85.0 (Shore)	1.14
**Si_3N_4**	15‘200 (Vickers)	3.18 - 3.4
**ZrO_2**	60‘000 (Vickers)	6.03
**Palladium**	100-190 (Vickers)	12

In order to avoid high abrasion, the vessel material had to be changed from typical, hard materials like zirconia and steel to custom made polymer vessels. Regarding the vessel, the polymer needed to meet certain properties. Firstly, it had to be resistant towards the chemicals used during the reaction. Secondly, the polymer needed to be hard enough to conduct the reaction upon collision with the milling ball since other impacts were excluded by the decision of using one ball. At a first glance, PMMA appears to be the most suitable material according to its hardness (cf. Table **S1**). However, PMMA tends to stress crack as soon as it is brought in contact with organic liquids. This property rules out numerous substrate combinations and PMMA was discarded as a vessel material. Polytetrafluoroethylene (PTFE) and polyfluoroalkoxy alkane (PFA) vessels were observed to meet the described criteria. Unfortunately, the PTFE balls and vessel underwent an aging process. Flakes of PTFE were formed in the reaction mixture, hindering the conversion after a couple of cycles, a process which does not happen for PFA (cf. Figure **S1 A** - **D**). Therefore, we decided to perform all experiments after the multiball screening with PFA vessels, as they are hard enough to conduct the reaction while featuring excellent anti adhesive behaviour and chemical resistance. The use of these milling tool combinations greatly decreased the abrasion to a level we at which not able to find any palladium abrasion in the raw product nor the final product using XRF.

2.2 Multiball approach

For the screening of multiple polymer balls together with one palladium ball, Polytetrafluoroethylene (PTFE), Polyoxymethylene (POM), Polypropylene (PP) were chosen as suitable materials for the additional balls. 1 - 3 Balls were added to the palladium ball in a 19 ml PFA vessel and were milled at 35 Hz for 1 h. The GC analysis shows clearly that only PTFE balls can be beneficial. However, as these balls are made of a PTFE shell with an iron core, those balls did not withstand the milling conditions and broke after few cycles. In the case of a broken PTFE ball, the iron core is set free and leads to major abrasion of the palladium ball, so even those balls were ruled out.

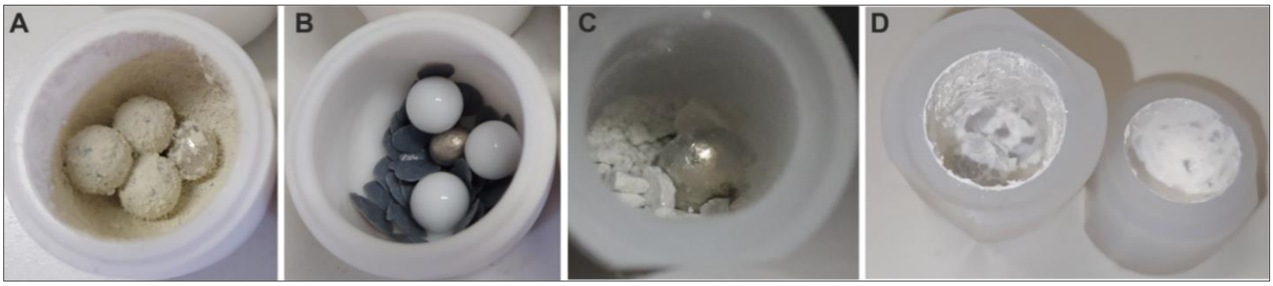

Figure S1: Pictures of several different approaches after milling. A) Multiball approach after one hour. The fine-grained mixture is evenly distributed in the vessel and on the milling balls. The conversion in this state was not quantitative, so the milling had to be continued B) Multiball approach after 16 h of milling. The PTFE flake formation is clearly visible. The conversion does not proceed as soon as the flake formation starts. C) One milling ball with K_2CO_3 and 1 mmol of each starting material without further additives after one hour of milling. A fine-grained raw mixture is observed. This approach yielded in 40 % product D) One milling ball with K_2CO_3 and 1 mmol of each starting material with LAG ethanol. The raw mixture appears to be a solidified melt. The sparkling substance at the walls of the vessel appeared to be the final product. The yield of this approach was 99%.

Table S2: Reaction conditions and yields of the model Suzuki coupling using different milling balls and vessels. One palladium ball in a polymer vessel was used with the addition of several balls from a different material (1 mmol of each substrate, 1 g K_2CO_3), 35 Hz, 1 h reaction time. Conversion in relation to remaining iodobenzene was measured**.**

Entry	Assisting balls	Vessel material	Abrasion	Conversion/Yield
**Suz-PTFE-3**	3 PTFE	PFA	14 mg	90 % / n.d.
**Suz-PTFE-1**	1 PTFE	PFA	<1 mg	0 % / n.d.
**Suz-POM-3**	3 POM	PFA	<1 mg	0 % / n.d.
**Suz-PP-3**	3 PFA	PFA	<1 mg	0 % / n.d.
**Suz-PA**[a]	none	PA-6	<1mg	99% / 99%[b]

[a] reaction conditions according to Table 1, Entry 1, iodine
[b] Yield determined by HPLC

2.3 Time dependent milling

To check for the necessary time until completion, time dependent milling experiments were done. The standard approach (cf. Table **1A**, Entry **1**) was milled in time increments up 6 hours in two different vessel types. After each period, the milling was stopped and a GC sample was taken out. These experiments showed that the time until complete conversion would be inacceptable.

Table S3: Reaction conditions and GC-MS-conversions of the Suzuki coupling model reaction after longer milling times. (1 mmol of each substrate, 1g K_2CO_3), 35Hz, varying milling time.

Entry	Abrasion	Milling time	conversion
**Suz-t1-1h[a]**	<1 mg	1 h	24 %
**Suz-t1-3h [a]**	**<1 mg**	**3 h**	**30 %**
**Suz-t1-4h [a]**	<1 mg	4 h	48 %
**Suz-t2-1h [b]**	**<1 mg**	**1 h**	**23 %**
**Suz-t2-3h [b]**	<1 mg	3 h	44 %
**Suz-t2-6h [b]**	**<1 mg**	**6 h**	**52 %**

[a] one Palladium ball in a 25ml PTFE vessel was used.
[b] one Palladium ball in a 18ml PTFE vessel was used.

2.4 Liquid-assisted grinding

During LAG screening, different polar liquids were tested for their applicability in liquid-assisted grinding. The standard approach (cf. Table **1A**, Entry **1**) was used for this screening with varying amounts of liquids (cf. Table **S4**).

Table S4: Reaction condition and GC-MS-conversions of the Suzuki coupling model reaction with various LAG additives and amounts. (1 mmol of each substrate, 1 g K_2CO_3), in a 19 ml PFA Vessel at 35 Hz, 1 h milling time.

Entry	Additive	η	Yield
**LAG-W-0.05**	Water	0.05	34 %
**LAG-W-0.1**	Water	0.1	14 %
**LAG-W-0.2**	Water	0.2	0 %
**LAG-B-0.15**	Beer[a]	0.05	70%[b]
**LAG-E-0.05**	Ethanol	0.05	77 %
**LAG-E-0.1**	Ethanol	0.1	80 %
**LAG-E-0.15**	Ethanol	0.15	99 %
**LAG-E-0.2**	Ethanol	0.2	70 %
**LAG-M-0.05**	Methanol	0.05	78 %
**LAG-M-0.1**	Methanol	0.1	73 %
**LAG-M-0.2**	Methanol	0.2	66 %
**LAG-I-0.05**	Isopropanol	0.05	63 %
**LAG-I-0.1**	Isopropanol	0.1	70 %
**LAG-I-0.2**	Isopropanol	0.2	64 %
**LAG-EG-0.05**	Ethylene glycol	0.05	12 %
**LAG-EG-0.1**	Ethylene glycol	0.1	23 %
**LAG-EG-0.2**	Ethylene glycol	0.2	3 %
**LAG-PEG-0.05**	Polyethylene glycol	0.05	28 %
**LAG-PEG-0.1**	Polyethylene glycol	0.1	34 %
**LAG-PEG-0.2**	Polyethylene glycol	0.2	17 %
**LAG-Ac-0.15**	Acetone	0.15	13%
**LAG-EtOAc-0.15**	Ethyl acetate	0.15	18 %

[a] Pils Beer of the Bochum Brewery Fiege was used, 5 Vol-% ethanol.
[b] Yield determinde by HPLC

2.5 Frequency dependence

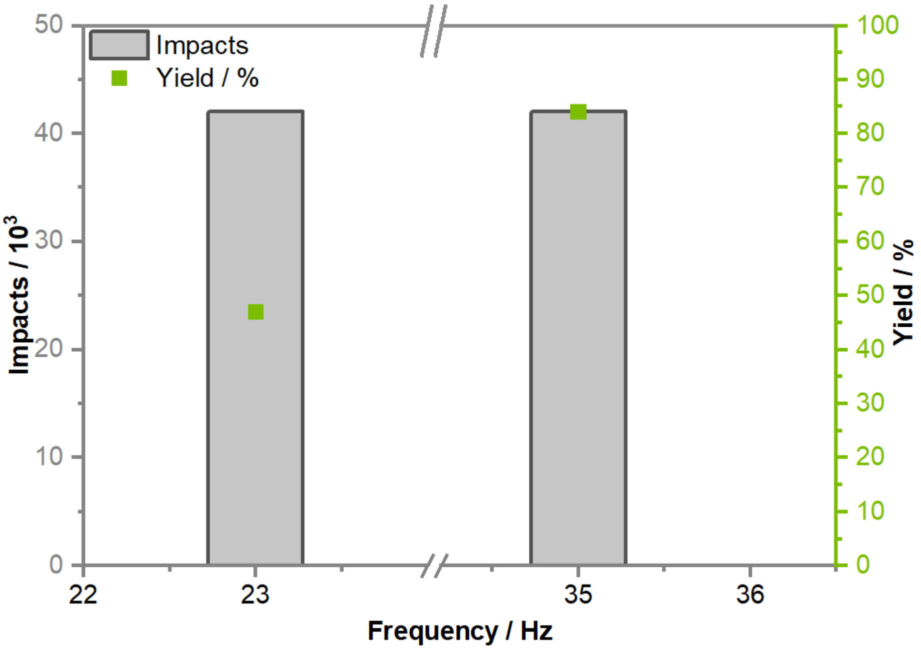

Figure S2: Frequency dependence of the Suzuki coupling of iodobenzene and phenylboronic acid using a 19 ml PFA vessel and a 10 mm Pd ball. The reaction was conducted with same amount of reactive collisions of the milling ball and the vessel, only differing in the frequency of milling. The higher milling frequency and thus, different ball motion, leads to different yield.

2.6 Influence of LAG on the 2,4,6-triphenylboroxin formation

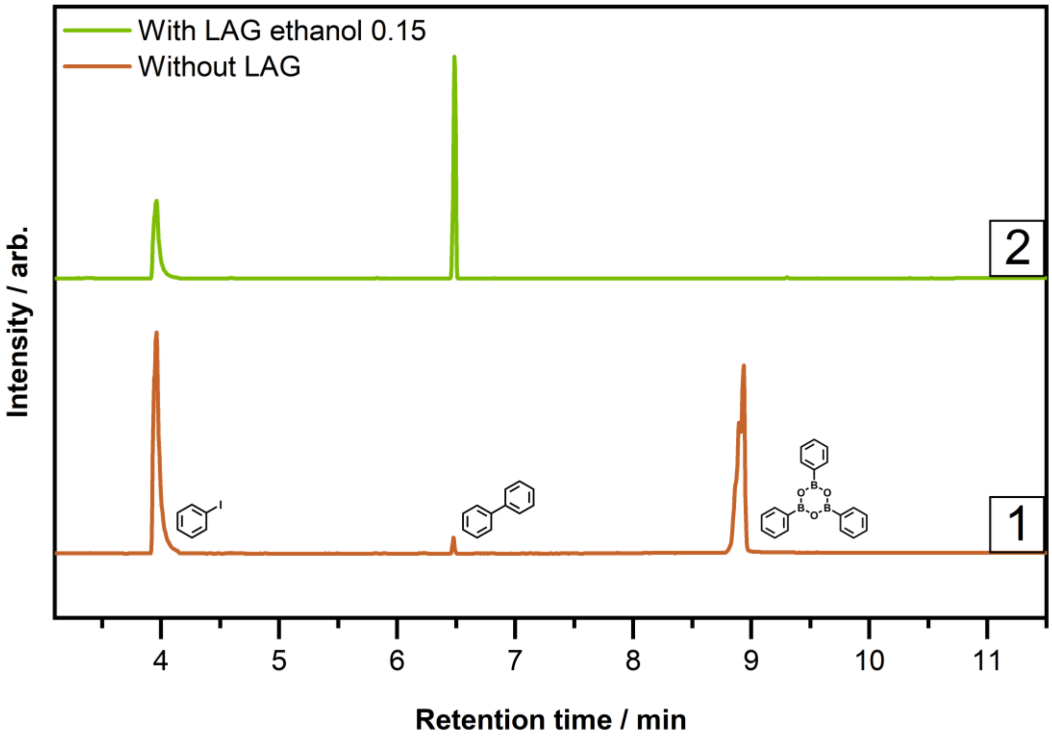

Figure S3: The influence of ethanol as a LAG additive on the formation of 2,4,6-triphenylboroxin. If no LAG additive is present, the boroxin is formed during milling (bottom chromatogram, peak at 8.9 min). This boroxin formation hinders the reaction as the small peak of biphenyl at 6.5 minutes indicate. As the reaction is hindered, a large iodobenzene peak is observable at 4 minutes. As soon as ethanol is added as LAG additive (top chromatogram), the boroxin is not formed, and the biphenyl peak becomes the major peak of the chromatogram, as the reaction to biphenyl is not hindered anymore.

2.7 Milling ball recycling

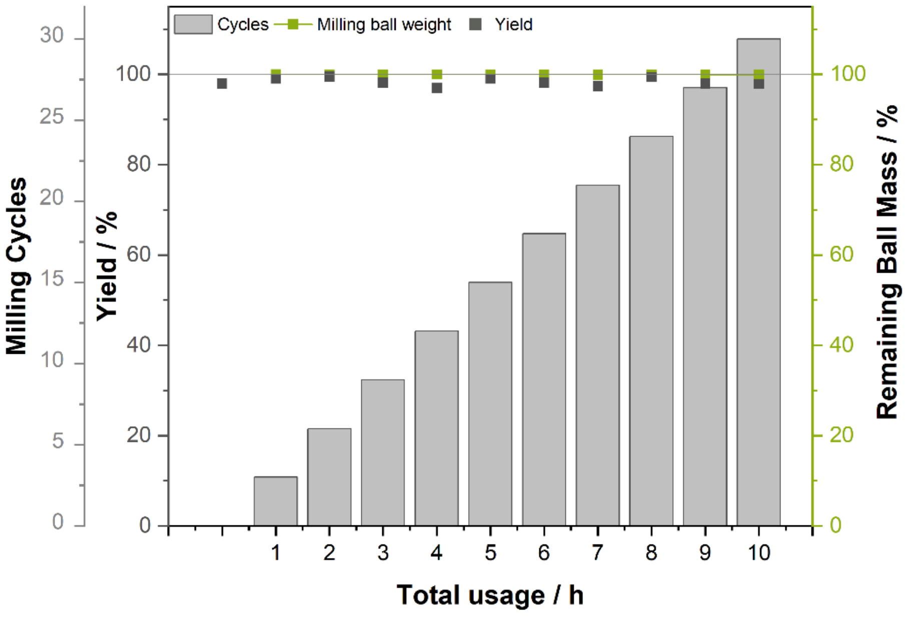

Figure S4: Milling ball weight over the course of multiple repetitions of the coupling between iodobenzene and phenylboronic acid. The milling ball weight remains constant even after 10h of milling time. The yield of each reaction remained constant.

3. Synthetic procedures

3.1 Direct mechanocatalytic Suzuki coupling

All experiments were carried out in the MM-500 mixer mill using PTFE and PFA milling vessels at 35 Hz for 1 h. If not stated otherwise, one single palladium milling ball was used.

General procedure for a Suzuki coupling using one milling ball: In a typical synthesis, 1 g of potassium carbonate was added to a 19 ml PFA vessel containing one palladium milling ball. On top of the potassium carbonate, 1 mmol of each starting material was added, followed by the respective amount of additive, if a reaction with KI or LAG was performed. The vessel was closed and milled for one hour at 35 Hz in a Retsch MM-500 vario mixer mill. After the milling, the milling ball was taken out and the mixture was transferred into water and extracted three times with 60 ml ethyl acetate each time. the solvent was evaporated and recycled for the next reaction, so the same ethyl acetate was reused during this screening. To remove insoluble substances and remaining phenylboronic acid, the raw product was passed through a small plug of silica in a Pasteur pipette using a Hexane/Ethyl acetate mixture (9:1). The solvent was evaporated and recycled as well. The final product was dried in vacuum.

General procedure for multiball approaches in the Suzuki coupling using several milling balls: In a multiball approach, a bigger vessel was used due to the higher space requirements of the additional balls. A 25 ml PFTE vessel was used for these approaches. As the volume of the vessel was enlarged, the amount of potassium carbonate was raised as well to ensure a sufficient mixing. 1.5 g potassium carbonate were added to the 25 ml PTFE vessel containing the palladium milling ball and the assisting balls, as described in table S2. The vessel was closed and milled for one hour at 35 Hz in a Retsch MM-500 vario mixer mill. After the milling, the milling balls were taken out and the mixture was transferred into water and extracted three times with ethyl acetate. The solvent was evaporated and recycled for the next reaction, so the same ethyl acetate was reused during this screening. To remove insoluble substances and remaining phenylboronic acid, the raw product was passed through a small plug of silica in a Pasteur pipette using hexane. The solvent was evaporated and recycled as well. The final product was dried in vacuum.

Procedure for catalyst leeching experiment: For catalyst leeching experiments, the standard approach was chosen. 1 g of potassium carbonate as well as 198 µL ethanol (LAG 0.15) were added to a 19 ml PFA vessel containing one palladium milling ball. Without any starting materials, the mixture was milled at 35 Hz for 10 and 60 minutes. after this time, the milling was stopped, the Pd ball was removed and exchanged for an inert zirkonia milling ball. The starting materials phenylboronic acid and iodobenzene were added and the milling was continued for the same time interval. After the milling, the mixture was transferred into water and extracted three times with ethyl acetate. The solvent was evaporated and recycled for the next reaction, so the same ethyl acetate was reused during this screening. From this crude product, a HPLC sample was taken.

3.2 Product characterization data and figures

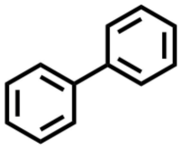

Biphenyl (Table 1, Entry 1)
1H NMR (400 MHz, Methylene Chloride-d2) δ 7.63 – 7.59 (m, 4H), 7.45 (dd, J = 8.4, 6.9 Hz, 4H), 7.38 – 7.33 (m, 2H).
EI-MS (*m/z*): [M+] calc. 154.21; found 154.10.

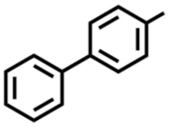

4-Methylbiphenyl (Table 1, Entry 2)
1H NMR (400 MHz, Methylene Chloride-d2) δ 7.62 – 7.56 (m, 2H), 7.53 – 7.48 (m, 2H), 7.43 (dd, J = 8.4, 6.9 Hz, 2H), 7.36 – 7.30 (m, 1H), 7.26 (d, J = 7.9 Hz, 2H), 2.39 (s, 3H).
EI-MS (*m/z*): [M+] calc. 168.24; found 168.

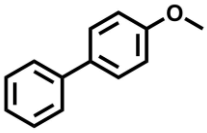

4-Methoxybiphenyl (Table 1, Entry 3)
1H NMR (400 MHz, Methylene Chloride-d2) δ 7.56 (td, J = 7.9, 7.0, 1.9 Hz, 4H), 7.42 (dd, J = 8.4, 7.0 Hz, 2H), 7.36 – 7.25 (m, 1H), 7.05 – 6.94 (m, 2H), 3.84 (s, 3H).
EI-MS (*m/z*): [M+] calc. 184.02; found 182.

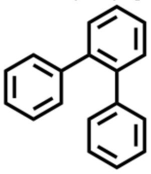

o-Terphenyl (Table 1, Entry 4)
1H NMR (400 MHz, Methylene Chloride-d2) δ 7.70 (dd, J = 7.4, 1.5 Hz, 2H), 7.47 (td, J = 7.6, 1.5 Hz, 2H), 7.40 – 7.30 (m, 10H).
EI-MS (*m/z*): [M+] calc. 230.31; found 230.20.

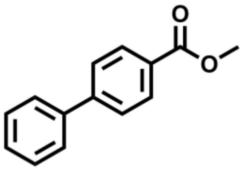

Methyl 4-biphenylcarboxylate (Table 1, Entry 5, I)
1H NMR (400 MHz, Methylene Chloride-d2) δ 8.09 (dd, J = 8.4, 1.6 Hz, 2H), 7.72 – 7.67 (m, 2H), 7.68 – 7.61 (m, 2H), 7.53 – 7.44 (m, 2H), 7.43 – 7.36 (m, 1H), 3.92 (s, 3H).
EI-MS (*m/z*): [M+] calc. 212.08; found 212.

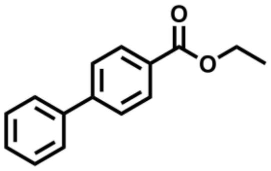

Ethyl 4-biphenylcarboxylate (Table 1, Entry 5, Br)
1H NMR (400 MHz, Methylene Chloride-d2) δ 8.09 (dd, J = 8.4, 1.6 Hz, 2H), 7.72 – 7.67 (m, 2H), 7.68 – 7.61 (m, 2H), 7.53 – 7.44 (m, 2H), 7.43 – 7.36 (m, 1H), 4.33 (q, 2H), 1.33 (t, 3H).
EI-MS (*m/z*): [M+] calc. 226.1; found 226.

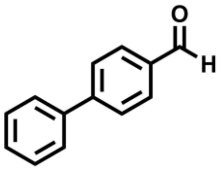

4-Biphenylcarbaldehyde (Table 1, Entry 6)
1H NMR (400 MHz, Methylene Chloride-d2) δ 10.05 (s, 1H), 8.03 – 7.91 (m, 2H), 7.82 – 7.75 (m, 2H), 7.72 – 7.63 (m, 2H), 7.49 (t, J = 7.5 Hz, 2H), 7.46 – 7.38 (m, 1H).
EI-MS (*m/z*): [M+] calc. 182.07; found 182.10.

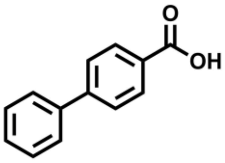

4-Biphenylcarboxylic acid (Table 1, Entry 7)
1H NMR (400 MHz, Methylene Chloride-d2) δ 8.20 – 8.15 (m, 1H), 7.92 – 7.77 (m, 2H), 7.76 – 7.71 (m, 2H), 7.70 – 7.65 (m, 1H), 7.54 – 7.46 (m, 2H), 7.41 (td, J = 7.3, 4.0 Hz, 1H).

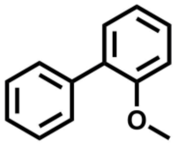

2-Methoxybiphenyl (Table 1, Entry 8)
1H NMR (400 MHz, Methylene Chloride-d2) δ 7.62 – 7.51 (m, 2H), 7.48 – 7.40 (m, 2H), 7.34 (dt, J = 5.5, 1.7 Hz, 2H), 7.10 – 6.96 (m, 2H), 3.83 (s, 3H).
EI-MS (*m/z*): [M+] calc. 184.09; found 184.

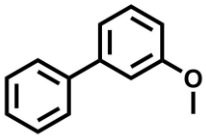

3-Methoxybiphenyl (Table 1, Entry 9)
1H NMR (400 MHz, Methylene Chloride-d2) δ 7.65 (ddd, J = 7.7, 2.6, 1.3 Hz, 2H), 7.48 (tt, J = 7.9, 1.8 Hz, 2H), 7.44 – 7.33 (m, 2H), 7.29 – 7.14 (m, 2H), 6.99 – 6.89 (m, 1H), 3.88 (t, J = 1.3 Hz, 3H).
EI-MS (*m/z*): [M+] calc. 184.09; found 184.

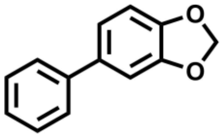

5-phenylbenzo[d][1,3]dioxole (Table 1, Entry 10)
1H NMR (400 MHz, Methylene Chloride-d2) δ 7.53 (d, J = 7.7 Hz, 2H), 7.41 (t, J = 7.6 Hz, 2H), 7.31 (t, J = 7.3 Hz, 1H), 7.09 (d, J = 7.3 Hz, 2H), 6.89 (d, J = 8.6 Hz, 1H), 6.00 (s, 2H).
EI-MS (*m/z*): [M+] calc. 198.07; found 198.

4. Mechanistic considerations

4.1 Determining the place of the catalysis

On several occasions, the milling balls' surface was claimed to be responsible for the catalysis.[1,2] However, at the same time numerous publications on Palladium nanoparticle catalysis exist[3]. It is imaginable that during the milling, Pd or PdO particles are abraded which in turn catalyse the reaction. This possibility must be accounted for as Pd remaining in the product are the main reason why these catalyses are used rarely in pharmaceutical synthesis. For the first time in the history of direct mechanocatalysis, we have the possibility to prove that the catalysis indeed proceeds on the milling ball surface and not within the mixture on microscopic particles, as we lowered the abrasion to a very low level. To prove our hypothesis, surface analysis of the milling ball, trace metal analysis of the milling mixture as well as leeching experiments were performed.

4.1.1 Leeching experiments

A straightforward approach to test the activity of abraded Pd in the Suzuki coupling for its catalytic activity would be to add Pd black to the reaction mixture and test for its catalytic activity. These experiments were already performed without success, indicating that Pd dust cannot be catalytically active.[1] However, the abraded Pd particles, which are suspected to conduct the catalysis, could be magnitudes smaller, in the range of nanoparticles. These nanoparticles would feature a completely different behaviour as nanoparticles are known for an astonishingly high catalytic activity.[3] To exclude these nanoparticles as the active catalyst, we had to generate the suspected particles in our milling setup before exposing the substrates to it. Therefore, a Pd milling ball of 4.3 g was milled with 1 g potassium carbonate and 198 µL (η 0.15) ethanol for a set time. The first milling duration was chosen to be 10 minutes as after this time, already 85 % yield could be achieved in the standard approach. After the milling of 10 minutes without substrates, the milling ball was removed and replaced by an inert milling ball made from zirconia before the substrates were weighted into the milling vessel. By this approach we can ensure that the starting materials can only be in contact to abraded Pd particles which in turn would be the only source for catalytic activity in this experiment. The milling was continued for 10 minutes. After this, the reaction mixture was treated in the typical manner by extraction and subsequent drying. HPLC analysis was performed from the raw product, revealing that only barely detectable traces were formed. To confirm these findings, the experiment was repeated and afterwards the milling time was prolonged to 60 minutes, both times leading to similar results.

In a second leeching experiment, all starting materials and the Pd milling ball were present from the start of the reaction. The milling was conducted for 2 minutes and a HPLC sample was taken. Subsequently, the milling ball was exchanged with an inert zirconia milling ball. After further 28 minutes of milling, the yield was determined gravimetrically and by HPLC. The experiment was conducted simultaneously to one reference experiment in which the Pd ball was exchanged by another Pd ball, to keep the amount of sample removed due to the ball swap comparable. HPLC analysis shows that in the case of the zirconia exchange experiment, the yield increases only slightly while the reference experiment shows a significant increase in conversion (cf. Figure **S5**). The slight increase in conversion when an inert milling ball is used, might either be due to accelerated aging processes[4] of activated starting material species or due to Pd complexes which might have formed during the milling in the reaction mixture. However, the percentage of converted substrates by this pathway is neglectable compared to the palladium ball like the previous experiment show. During these experiments, the yield of the Suzuki coupling remains lower than in an experiment with uninterrupted milling. This is likely due to rheological effects, as the reaction mixture becomes a paste during milling which solidifies during the ball exchange. This solidification hinders the conversion at first until the previous rheological state is achieved again (cf Figure **S5**). We thereby can confirm that the major catalytically active species is not the abraded palladium nanoparticles but the palladium milling balls.

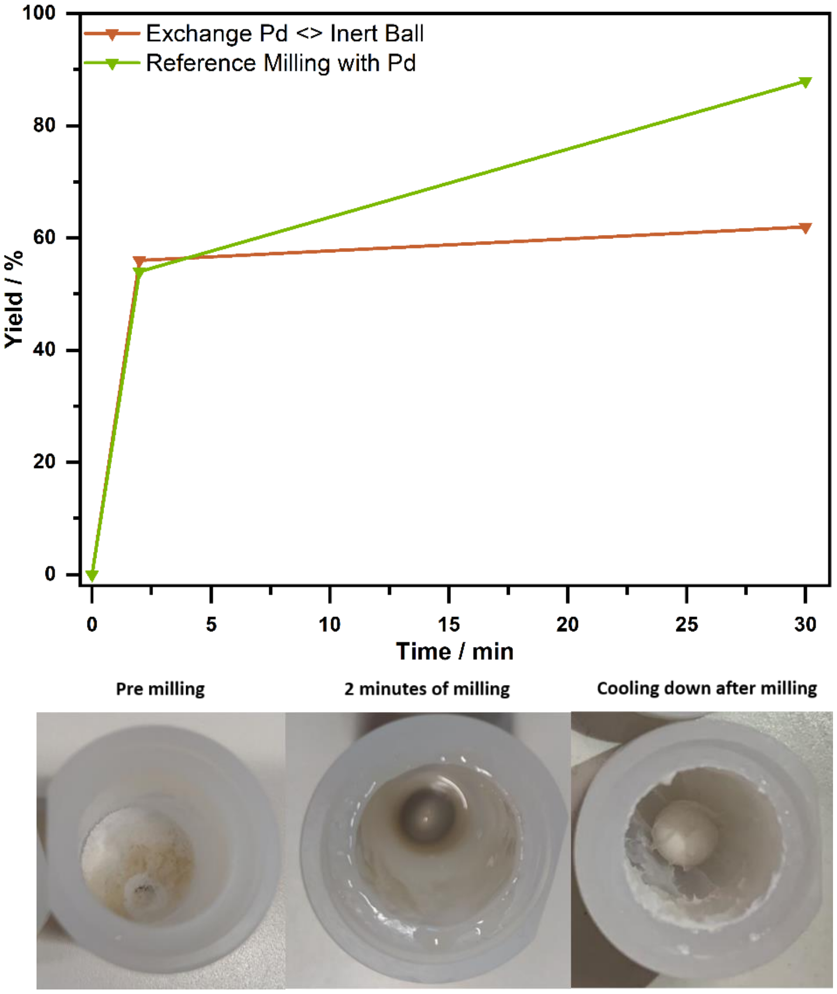

Figure S5: Second leeching experiment and pictures of the milled mixtures. The reaction mixture was milled for 2 minutes using a Pd milling ball. After these two minutes, the catalytically active milling balls got exchanged by an inert zirconia milling ball. After the exchange, no increase in yield is detectable. A reference experiment in which the Pd ball remained in the milling vessel shows an increase in yield. The slower increase is due to rheological reasons, as the milling mixture, which is a paste after the first two minutes of milling, solidifies during the procedure of exchanging balls as shown in the pictures. Therefore, the yield does not increase with the same speed after the milling is continued.

4.1.2 X-ray photoelectron spectroscopy

After we could prove that the catalyst is not generated during the milling in the form of microscopic particles, we wanted to examine the milling ball for indication of the nature of this catalysis. To gain information about the catalytic cycle, XPS analysis of the milling ball was performed. Three measurements were done. First, a thoroughly cleaned Pd milling ball which was used before was analysed, showing a clear signal of Pd(0) indicated by the signals at 335.4 and 336.7 eV, respectively (cf Figure **S6 A**). Next, a clean Pd milling ball was milled for one hour with only iodobenzene. After drying the milling ball at room temperature in vacuum, XPS analysis was performed (cf Figure **S6 B**). A significant difference can be seen between these two measurements. Apparently, during the milling, a Pd(II) species, indicated by the signals at 336.7 and 340.2 eV, is formed upon contact to iodobenzene, comparable to the oxidative addition step in homogeneous Palladium catalysis. These results are in line with the Literature.[5] The existence of this Pd species proves that the catalysis indeed proceeds on the milling ball surface, as the cleaved aryl iodide is located on the milling ball surface, where it can react with its counterpart, the phenylboronic acid. The milling plays an important role in this oxidative addition, as a Pd ball, which was covered in iodobenzene and measured without any application of thermal or mechanical energy, shows only the Pd(0) signal observed on a clean Pd ball. A thorough investigation of a standard approaches (cf Figure **1** A, Entry **1**) milling mixture did not show any Pd signal (cf Figure **S6 D**).

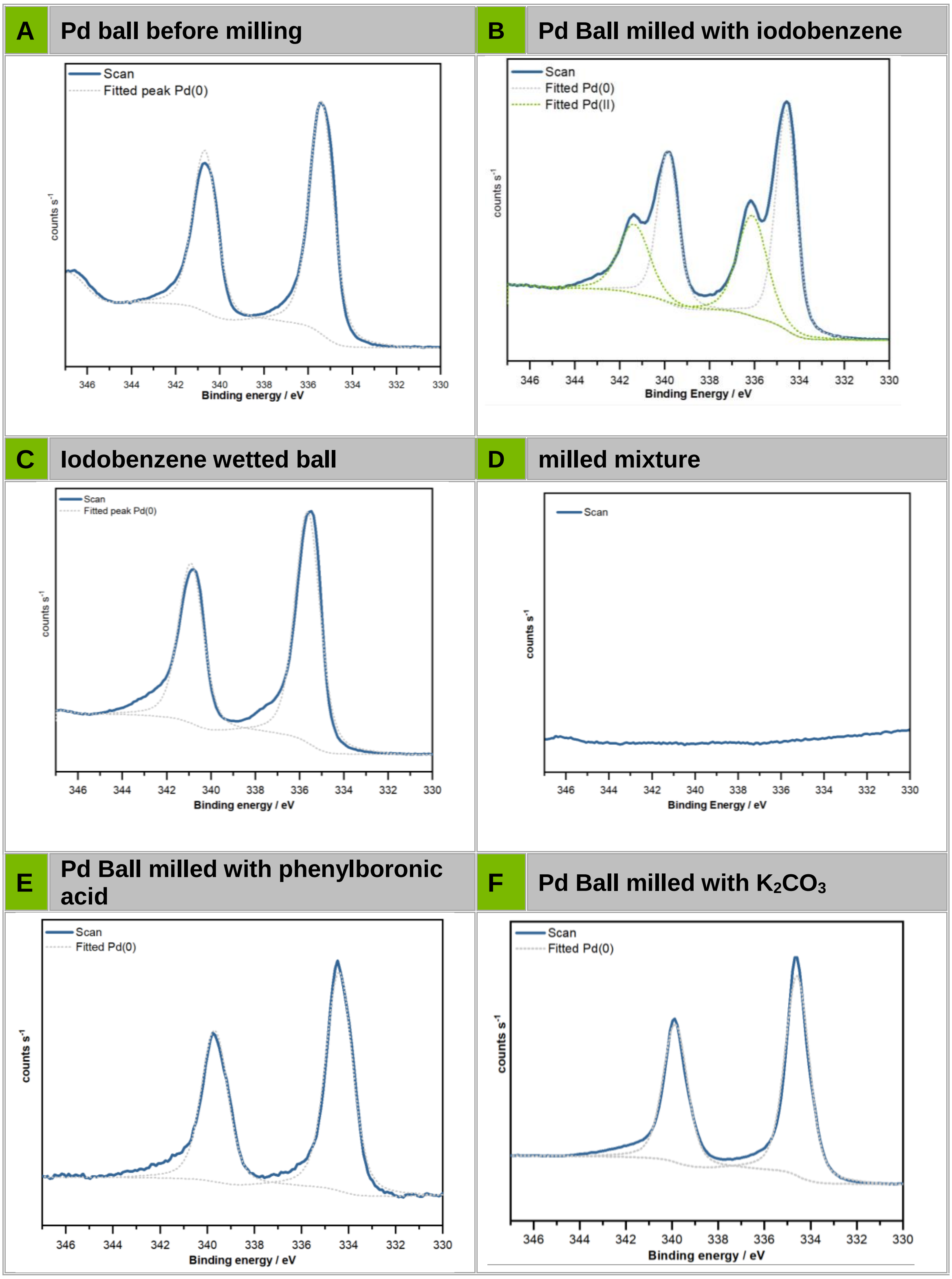

Figure S6: XPS analysis of milling balls under various conditions. **A)** A clean Pd ball was measured. Only the signals of a Pd(0) species is to be found. **B)** XPS spectrum of a Pd milling ball after milling the ball with iodobenzene for 30 minutes. Two signals appeared which were not present in the spectrum of the clean milling ball. These two signals resemble a formed Pd(II) species, formed after the oxidative addition of iodobenzene. **C)** XPS spectrum of a Pd milling ball which was coated in iodobenzene without application of thermal or mechanical energy. No Pd(II) signal can be found as opposed to spectrum B. **D)** XPS spectrum of a milled mixture. No Pd signal can be found. **E)** Clean Pd ball milled for 30 minutes with potassium carbonate and ethanol. Notably, no new signals can be found, leading to the conclusion that phenyboronic acid cannot interact with a Pd surface without prior oxidative addition of iodobenzene **F)** Pd milling ball milled for 30 minutes with potassium carbonate. no new signals can be found**.**

4.1.3 Scanning electrode microscopy and Energy-dispersive X-ray spectroscopy

The milling balls and raw mixtures were subsequently analyzed by SEM and EDX. We could confirm the findings of XPS spectroscopy. After milling of a Pd ball with iodobenzene, the surface, previously only containing Pd, shows a carbon layer, indicating the adsorbed species after oxidative addition (cf. Figure **S7**). Large Pd flakes which might have been abraded during the milling and which in turn could be too big to be dissolved and analyzed by ICP-OES could not be found in any reaction mixture (cf Figure **S8**).

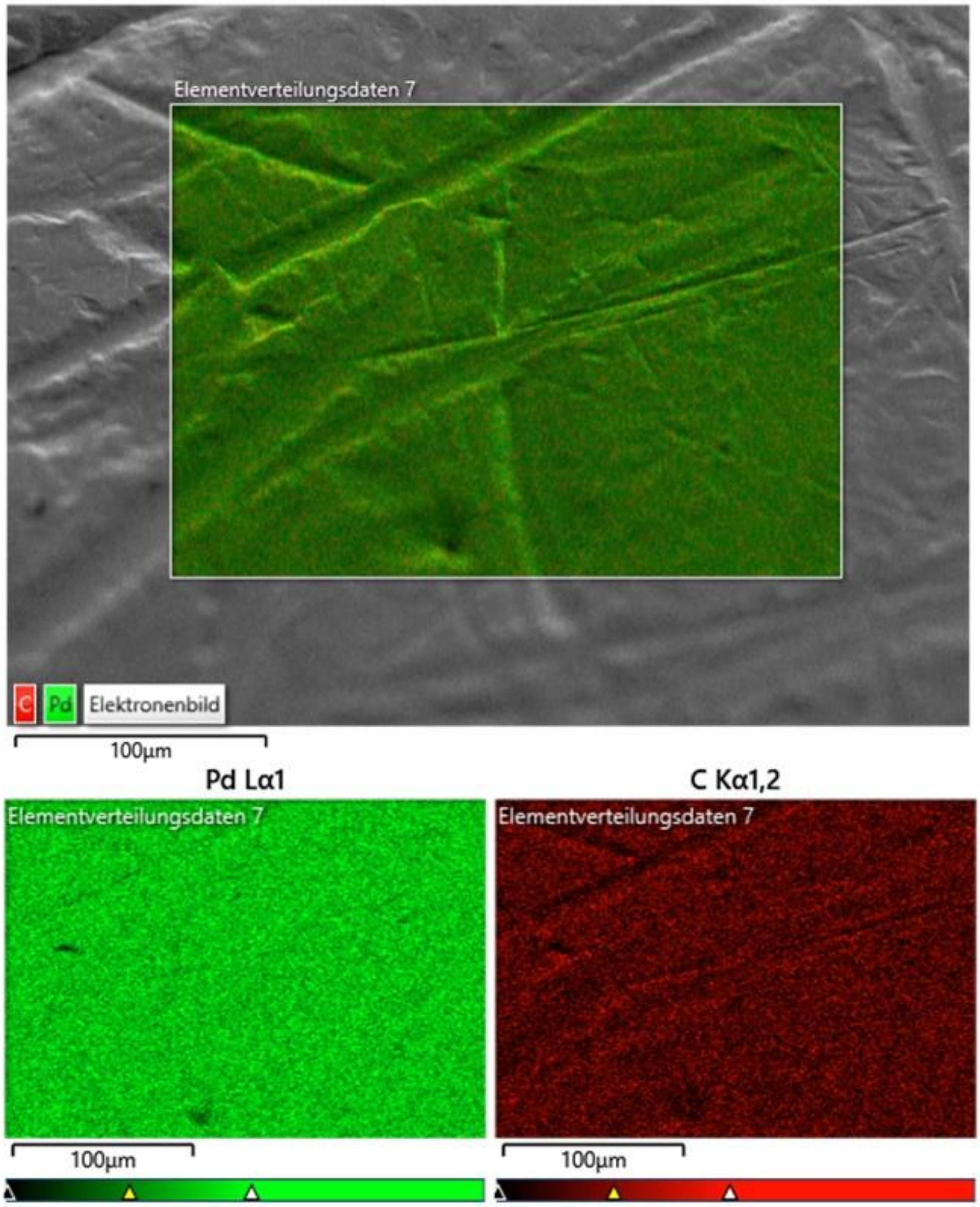

Figure **S10**: SEM and EDX analysis of a Pd milling ball after being exposed to Iodobenzene. Even though the milling ball was dried in high vacuum, carbon is still present on the milling ball surface, confiriming the findings of XPS analysis.

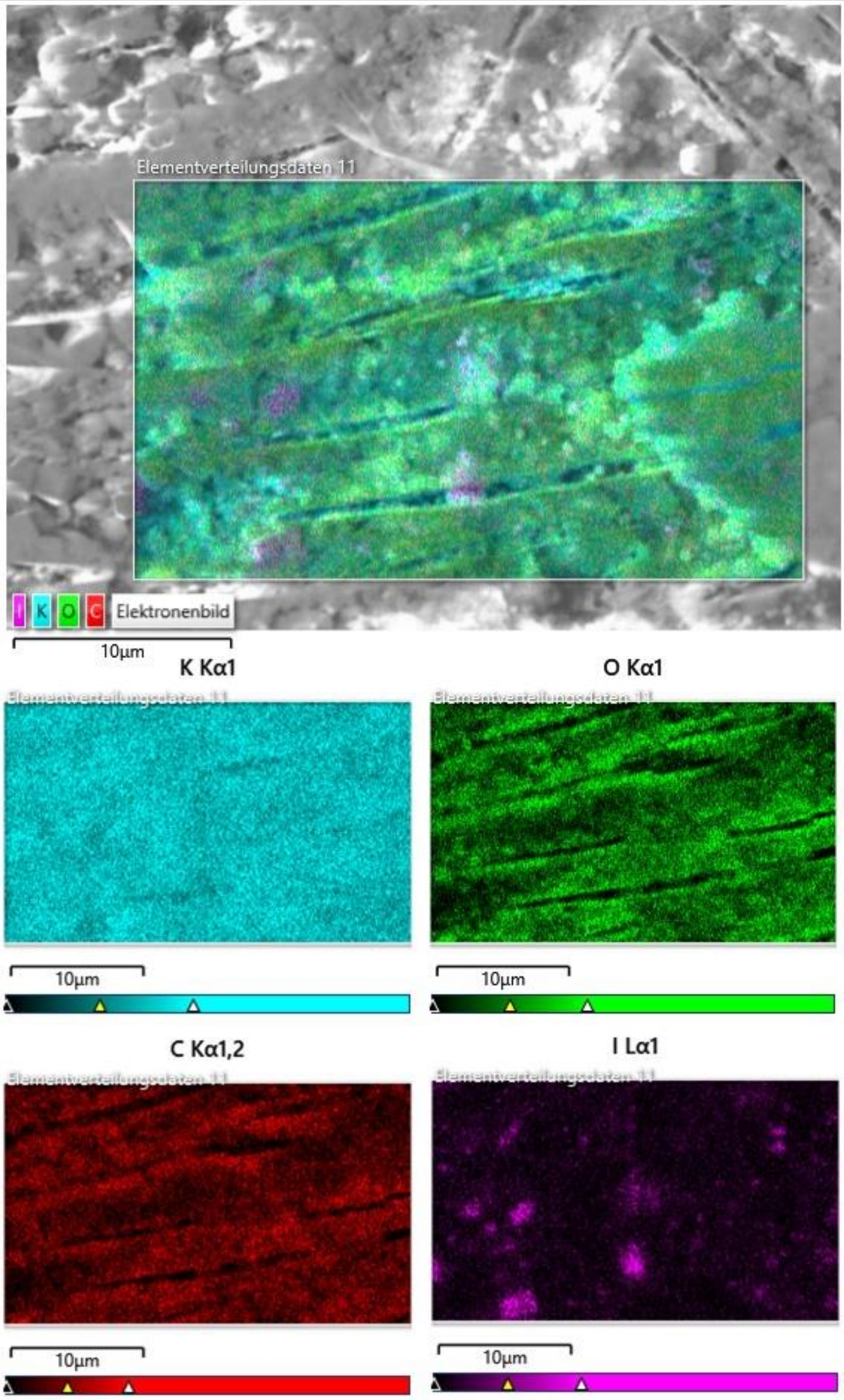

Figure **S8**: SEM and EDX analysis of a reaction mixture after 60 minutes of milling. Only carbon, potassium, oxygen and iodine can be found, resembling the starting materials. No Pd flakes can be detected.

4.1.4 Inductively coupled plasma atomic emission spectroscopy

To determine the exact amount of Palladium which is to be found in the milling mixture and the final product, ICP-OES analysis was performed. Each a sample of a milling mixture and a purified product were analysed.

Table S10: Results of ICP-OES analysis of a milled mixture prior to workup and the final product after extraction and microcolumn chromatography.

Pd content milled mixture [ppm]	Pd content final product [ppm]
0.6715	0.471

These results show that during 60 minutes of milling, $8.91 * 10^{-7}$ g (891 nanogram) Pd are abraded and transferred into the whole reaction mixture. Remaining Pd in the final product is significantly below the limit for pharmaceutical application.[6]

4.2 In situ XRD

To understand the nature of the elaborated conversion, *in situ* XRD studies were made. First, we wanted to find out whether the reaction starts immediately upon shaking of the milling vessel or if the reaction needs a certain incubation time until the product is formed. For this first examination, the standard reaction was chosen (cf. Figure **1A**, Entry **1**). Unfortunately, the starting materials are not trackable under the conditions the PXRD measurements were performed. However, we could observe the nature of this coupling by investigating the reflex of the side product, which is potassium iodide. As the formation of this salt is bound to the formation of the biphenyl product, the *in situ* XRD measurements can be used for our purpose. This first measurement already gave us insight into the reaction. As shown in figure **S9 A**, the product formation does not start immediately after the mill is started. After 3 minutes, the product formation starts noticeably and increases drastically. This indicates that the surface of the lone palladium milling ball needs to be occupied by the starting material before the coupling can occur. These results explain why solid starting materials react slower than other, liquid, materials, such as 4-iodotoluene or methyl-4-iodobenzoate because liquid starting materials are more readily distributed over the surface of the milling ball. Moreover, understanding that the reaction speed is linked to the ability of the starting material to accumulate on the milling balls surface, we can explain why ethanol is such a powerful LAG additive. This first measurement provided valuable insight into the catalytic nature of direct mechanocatalysis, as the palladium ball acts like a heterogeneous catalyst. In following experiments, we wanted to examine our elaborated parameters for this coupling even further. First, we wanted to check for the substrate scope by using halogen aromatic compounds with either an iodine, bromine or chlorine halogen atom. To be able to observe the starting materials consumption and simultaneous product formation, halogenated benzaldehyde derivatives were chosen as they react readily in the case of 4-iodobenzaldehyde and are solid even in the case of 4-chlorobenzaldehyde. We could track the starting material consumption and subsequent start of product formation using these systems. As expected for the Suzuki coupling, the hierarchy of reactivity was I > Br (cf. Figure **S9 B-C**). To ultimately find out about the applicability of our method to chlorinated substrates, a reaction of chlorobenzene and phenylboronic acid was investigated. We chose chlorobenzene over chlorobenzaldehyde due to the previously observed effect of liquid starting materials reacting faster. Soon after the milling was started and even before the added KI was consumed, we could observe a new reflex in the diffractogram (cf. Figure **S9 D**). In this case, a phenylboronic homocoupling proceeded as no suitable arylhalogen was present to react with the phenylboronic acid, which means the new reflex is caused by a boronic acid compound. This behaviour was observed earlier and led to a yield of biphenyl of roughly 10% after 1h of milling. We could rule out the phenylboronic acid homocoupling as a major side reaction in other couplings, as this formation was not observed if a sufficiently reactive, substituted arylhalogen was used in the coupling. In the cases of 4-iodoanisol or 4-iodobenzaldehyde as substrate for example, no biphenyl could be found in GC-MS. To further evaluate the impact of additives, the reaction of 4-bromobenzaldehyde and phenylboronic acid was tested once with the addition of 10 mol-% KI and one time without said addition. To our delight, our observation that potassium iodide enhances the yield of the coupling of aryl bromine compounds was confirmed. *In situ* XRD analysis shows clearly the disappearance of potassium iodide. Upon consumption of KI, the product formation starts, indicating that a trans halogenation took place which enhances the yield of the conversion (cf. Figure **S9 C**). However, it appears that the onset of product formation ultimately is hindered. This might be the case because bromoaryl compounds have to react on the palladium surface with the offered KI first.

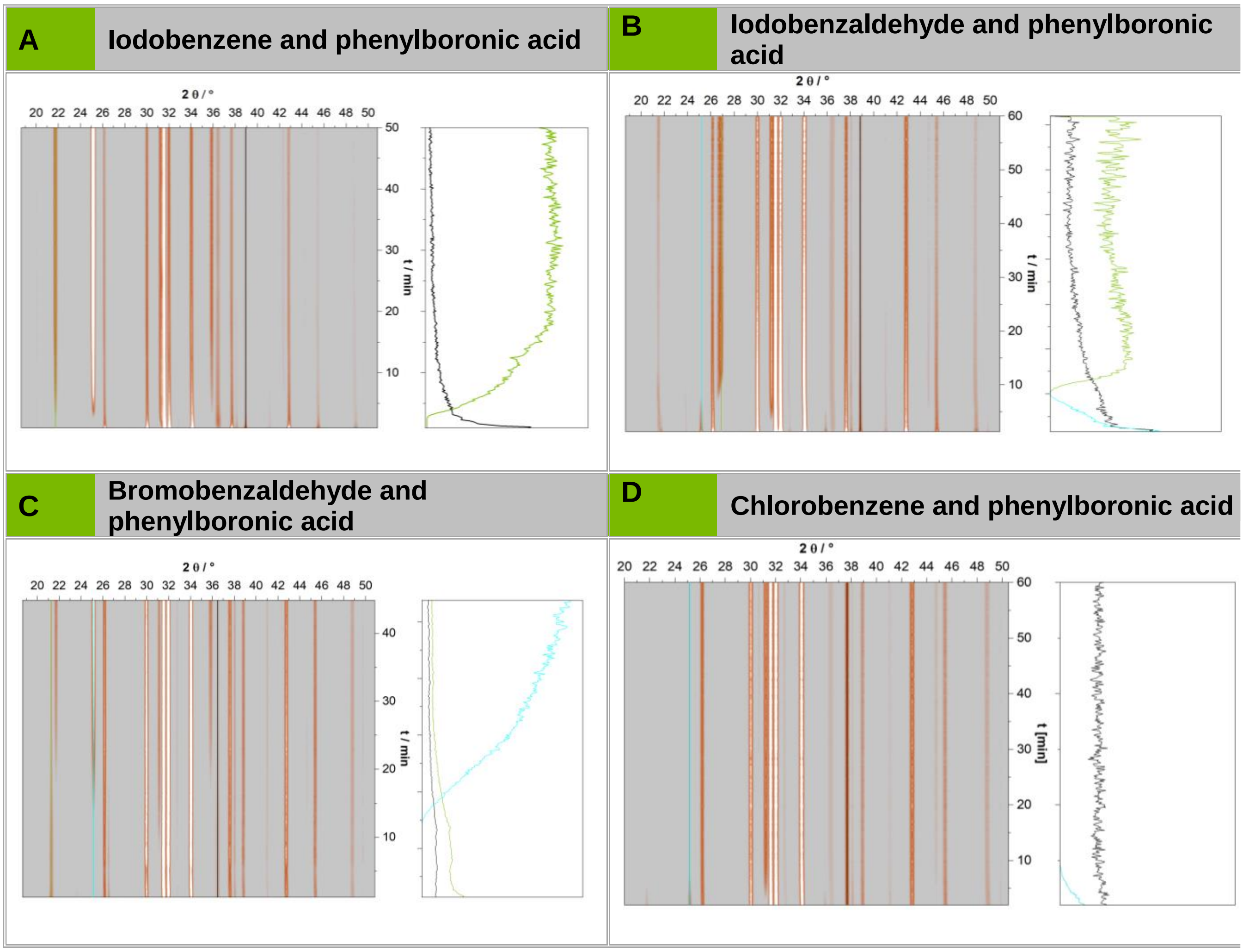

Figure S8: Different in situ XRD measurements depending on the halogen atom. Black lines show potassium carbonate, blue lines show starting materials and mixtures, green lines highlight the new reflexes after the product is formed. When potassium iodide is not used as the main indicating reflex, it is highlighted as light blue line. **A)** In situ PXRD analysis of the coupling between iodobenzene and phenylboronic acid. The reflex of the potassium iodide (green line) at 25 ° starts forming after 3 minutes and gains intensity very quickly. Simultaneously, the reflex of potassium carbonate, shown as a black line at 37.6 ° decreases. **B)** Suzuki coupling of 4-Iodobenzaldehyde and phenylboronic acid. The product formation starts after an incubation time of roughly 20 minutes at 21.7 ° (green line) while the starting material reflex at 21.2 ° decreases. Simultaneously, the formation of potassium iodide (light blue line) is observable. **C)** Suzuki coupling of 4-bromobenzaldehyde and Phenylboronic acid. The consumption of added KI before the formation of new reflexes at 21.7 ° is clearly visible. The incubation time which was 20 minutes for iodobenzaldehyde now surpasses 50 minutes. **D)** Suzuki coupling of chlorobenzene and phenylboronic acid. During this reaction, a new reflex is formed after only 3 minutes of milling time and even before the KI is consumed. No Biphenyl formation was observed in *ex situ* analysis.

4.3 Raman investigations on the phenylboronic ester formation

The assumption of a phenylboronic ester, which is formed during the reaction was checked by Raman spectroscopy. Therefore, a standard approach without iodobenzene was milled for an hour. As LAG additives, water and ethanol were added to the phenylboronic acid. The raw mixture was washed with ethyl acetate to collect all organic substances and the mixtures were thoroughly dried in a vacuum drying oven at 80°C to removeany remaining liquids. Raman spectroscopy of the raw mixtures was performed *ex situ*, proving the assumption of the alcohol forming the ester during milling (cf. Figure **S11**).

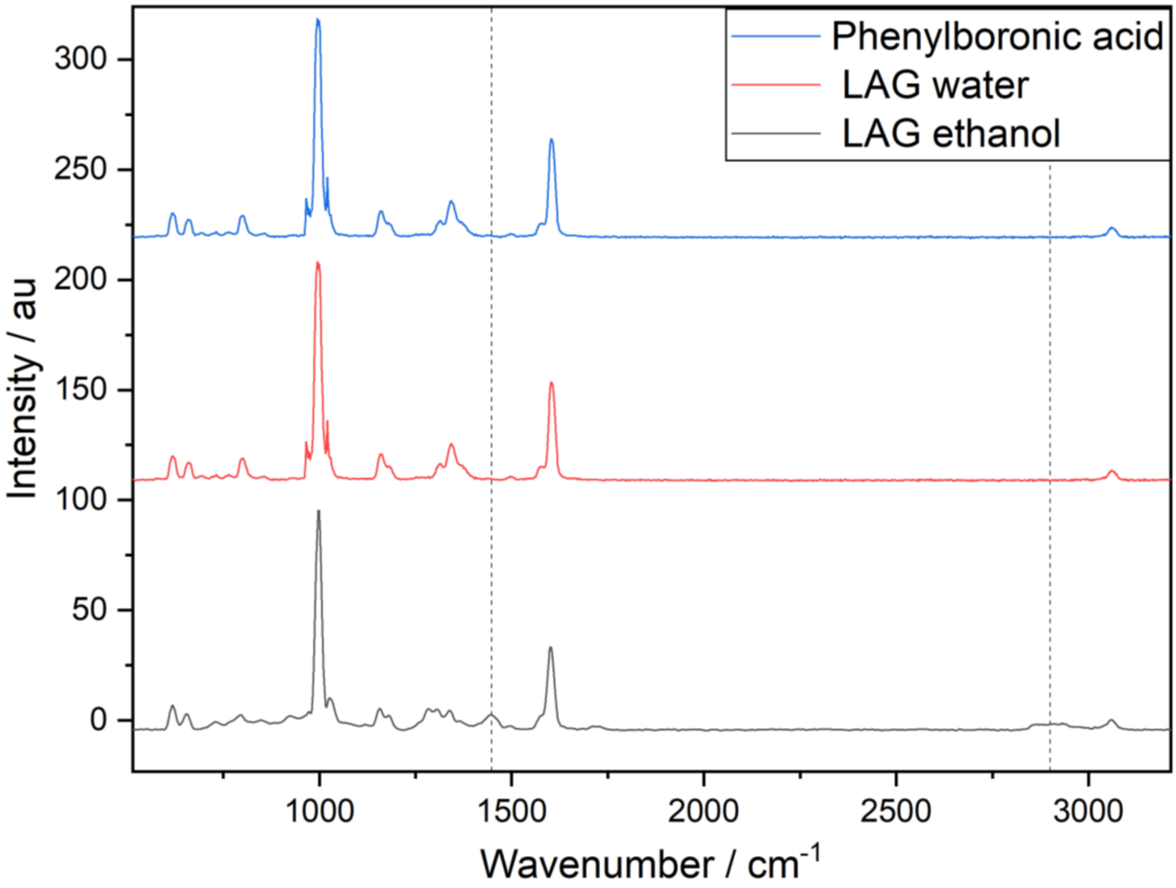

Figure **S11**: Raman spectra of two reference experiments to prove the ethyl ester formation. Phenylboronic acid was milled without iodobenzene with potassium carbonate and the respective LAG additive. It can be seen that in the case of water as a LAG additive, no change compared to the reference spectrum of phenylboronic acid is visible. in the case of ethanol as LAG addtive, new bands appear. The most significant ones at 1448 cm^{-1} and at 2963 cm^{-1} to 2839 cm^{-1} indicate the formation of the ethyl ester, as those bands represent aliphatic C-H vibrations.

4.4 Detailed calculations on monolayer hypothesis

To determine whether it is possible that on each impact a monolayer on the milling ball reacts to the desired product, simplified calculations were done. We assumed for our calculations that each starting material has the size of benzene and mass transport phenomena to and from the catalyst ball surface are negligible. As in reality, the starting materials are considerably bigger than benzene and that indeed mass transport is a hindering factor, this assumption is idealized in favour of the monolayer theory. Essentially, the surface demand of 2 mmol of benzene were calculated and divided by the catalyst ball surface. This fraction should then represent the necessary hits of the milling ball to achieve full conversion if on every impact, a perfectly covered milling ball surface reacts completely to the desired product. As the resulting theoretically required amount of hits still exceed the actually required amount of hits by the factor of 5.95, we concluded that other factors have to be considered to explain the fast conversion, being the theory that a film is formed around the ball, providing a diffusion-enabling boundary layer.

Surface of the milling ball:

$$A_{Ball} = 4 * \pi * r^2 \ = 314.16\, mm^2$$

Molecules in 2 mmol benzene:

$$n = N_A * n = 6{,}022 * 10^{23} * 0{,}002 = 1{,}20 * 10^{21}$$

Surface demand of 2 mmol Benzene:

$$\pi * r_{Benzene}{}^2 = (2.495\, mm * 10^{-7}\,)^2 = 1.955 * 10^{-13}\, mm^2$$

Number of Benzene molecules which fit on the Ball Surface in a monolayer:

$$n_{Monolayer} = \frac{A_{Benzene}}{A_{Ball}} = \ 1.60 * 10^{15}$$

Amount of hits required to achieve full conversion:

$$\frac{n}{n_{Monolayer}} = 749'742$$

Experimentally determined required collisions for full conversion:

$$v * t * 2 = 35\ \frac{1}{s} * 1800\,\mathrm{s} * 2 = 126'000$$

4.5 Transhalogenation

In harmony with the literature, a trans halogenation by milling brominated compounds with potassium iodide was attempted. The formed Iodobenzene could be tracked in GC analysis if the milling was stopped before the conversion was complete.

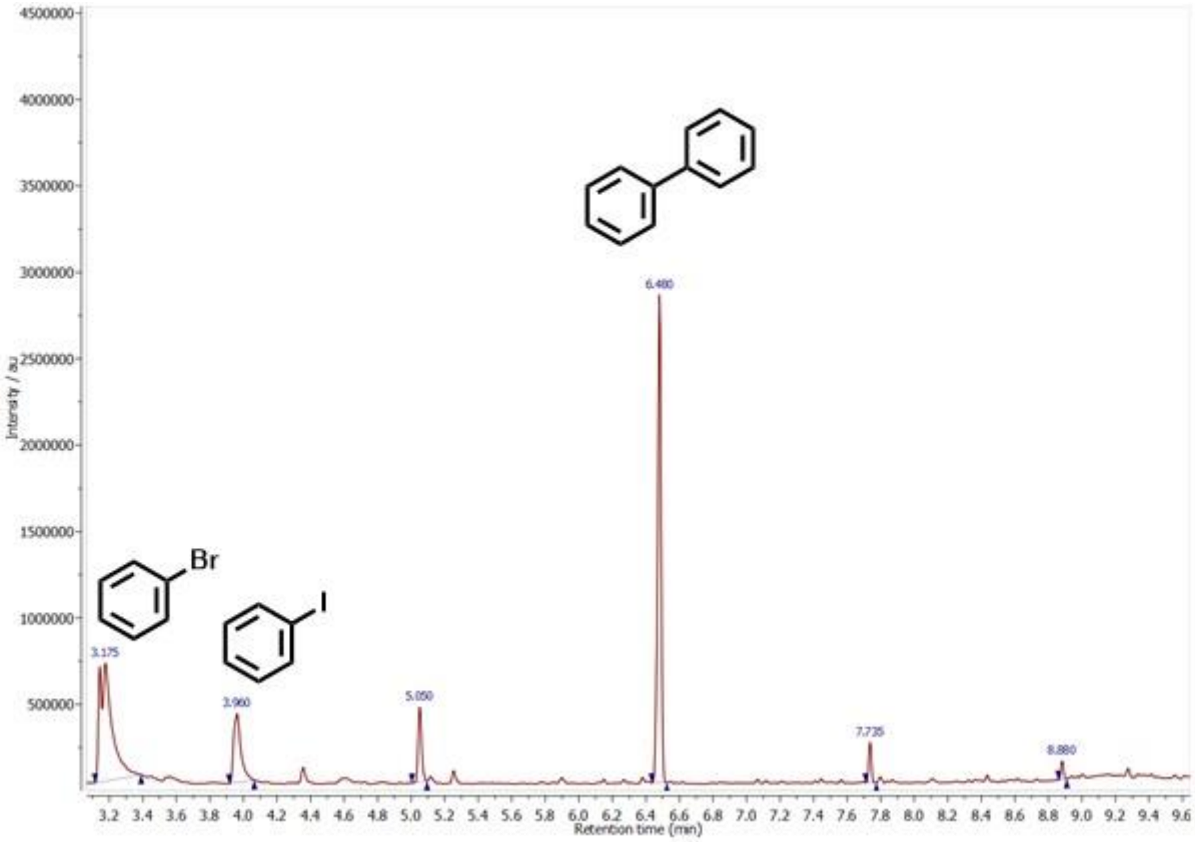

Figure S12: Gas chromatogram of a reaction featuring bromobenzene and potassium iodide. The Peak at 3.175 min shows bromobenzene which is partially converted into iodobenzene, seen at 3.96 min. The reaction is partially complete, as the big peak at 6.480 min shows

4.6 Cross-coupling validation

To validate the nature of the direct mechanocatalytic coupling being a cross-coupling experiments were performed utilizing starting materials which would lead to an asymmetric product. Additionally, this experiment was further utilized to verify the state of the milling ball during and after the reaction by XPS analysis. As starting materials, iodobenzene and 4-methoxyphenylboronic acid were chosen. Similar to the experiments shown in chapter 4.1.2, the milling ball was pre-milled with iodobenzene and XPS analysis was performed on its surface, before the milling ball was returned to the reaction mixture containing the boronic acid, ethanol and the base. Afterwards the milling was continued an additional 10 minutes, the milling ball was removed from the reaction mixture and again analysed by XPS, while the reaction mixture was investigated by GC-MS for the formed product. In line with the previous results, the milling ball shows a Pd(II) species during the reaction, which is not detectable on the milling ball after the reaction is completed. In the reaction mixture, only the cross-coupling product 4-methoxybiphenyl can be found, proving that no homocoupling occurs.

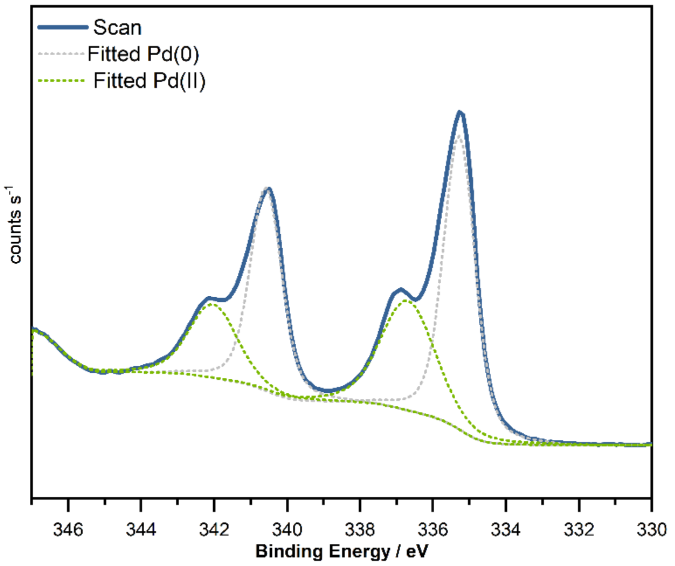

Figure S13: XPS spectrum of a Pd ball after milling with iodobenzene. The Pd(II) signals caused by the oxidative addition are to be seen as shoulders of the main Pd(0) signal.

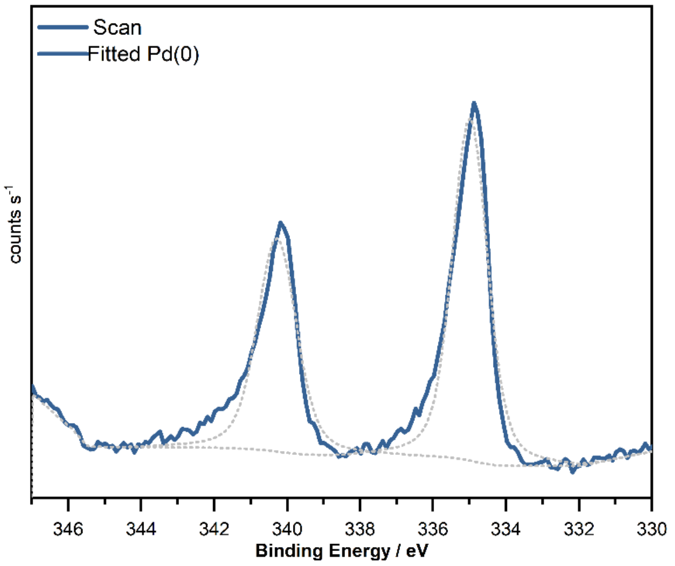

Figure S14: XPS spectrum of the Pd milling ball after the successful coupling. only Pd(0) signals are detected in this spectrum, as previously seen oxidatively added species were reacted with 4-methoxyphenylboronic acid.

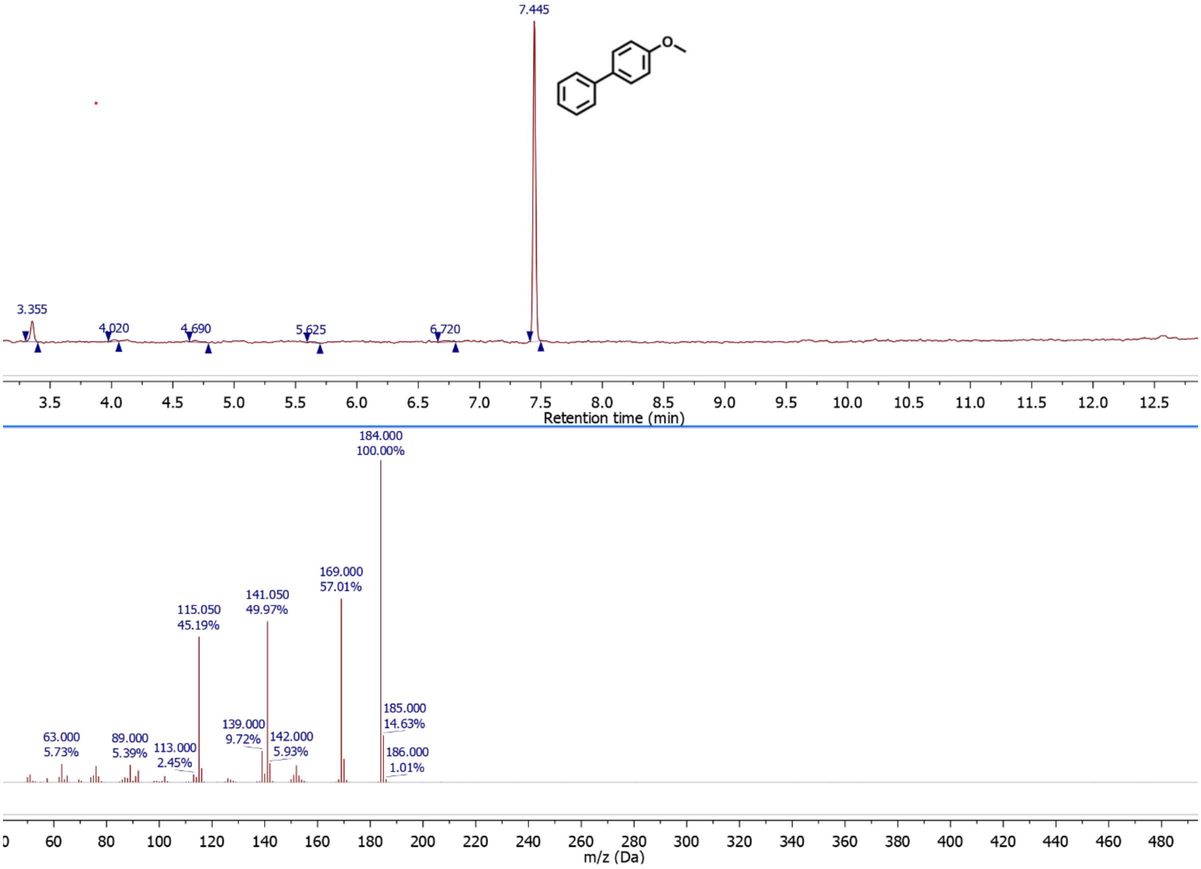

Figure S15: GC-MS results of the coupling of 4-methoxyphenylboronic acid and iodobenzene. Only methoxybiphenyl can be detected, proving the cross-coupling nature of this protocol. If a homocoupling of the boronic acid or the aryl halide would occur, biphenyl or 4,4'-dimethoxybiphenyl would be present.

Author contributions

**Wilm Pickhardt**	Preparation of the manuscript, mechanochemical reactions and sample purifications, XPS analysis, Synchrotron XRD, HPLC analysis, ICP-OES sample preparation, GC-MS and Raman measurements
**Claudio Beaković**	Mechanochemical reactions, sample purification, sample analysis
**Maike Mayer**	Mechanochemical reactions, sample purification, sample analysis
**Maximilian Wohlgemuth**	XPS sample preparation, analysis and postprocessing
**Fabien Joel Leon Kraus**	Mechanochemical reactions, sample analysis
**Sven Grätz**	Preparation of the manuscript, supervision
**Lars Borchardt**	Project coordination and supervision

[1] C. G. Vogt, S. Grätz, S. Lukin, I. Halasz, M. Etter, J. D. Evans, L. Borchardt, *Angew. Chem. Int. Ed.* **2019**, *58*, 18942.
[2] a) C. G. Vogt, M. Oltermann, W. Pickhardt, S. Grätz, L. Borchardt, *Adv. Energy Sustain. Res.* **2021**, *2*, 2100011; b) W. Pickhardt, S. Grätz, L. Borchardt, *Chem. - Eur. J.* **2020**, *26*, 12903.
[3] M. Pérez-Lorenzo, *J. Phys. Chem. Lett.* **2012**, *3*, 167.
[4] T. Friščić, C. Mottillo, H. M. Titi, *Angew. Chem., Int. Ed.* **2020**, *59*, 1018.
[5] a) Yamada, Masuda, Park, Tachikawa, Ito, Ichikawa, Yoshimura, Takagi, Sawama, Ohya et al., *Catalysts* **2019**, *9*, 461; b) A. M. Venezia, *Catalysis Today* **2003**, *77*, 359.
[6] S. Phillips, D. Holdsworth, P. Kauppinen, C. Mac Namara, *Johnson Matthey Technology Review* **2016**, *60*, 277.
